# A randomized trial of adapted versus standard versions the transdiagnostic intervention for sleep and circadian dysfunction (TSC) implemented via facilitation and delivered by community mental health providers using train-the-trainer

**DOI:** 10.1186/s13012-025-01467-y

**Published:** 2025-11-29

**Authors:** Allison G. Harvey, Emma R. Agnew, Rafael Esteva Hache, Catherine A. Callaway, Estephania Ovalle Patino, Anne Milner, Julia M. Spencer, Marlen Diaz, Lu Dong, Amy M. Kilbourne, Daniel J. Buysse, Eric Stice, Laurel D. Sarfan

**Affiliations:** 1 Department of Psychology, University of California, 2121 Berkeley Way, Berkeley, CA 94720 USA; 2 RAND Corporation, Santa Monica, CA USA; 3 Office of Research and Development, Veterans Health Administration, U.S. Department of Veterans Affairs, Washington, MI USA; 4 University of Pittsburgh, Pittsburgh, PA USA; 5 Stanford University, Stanford, CA USA; 6 Department of Research and Evaluation, Kaiser Permanente Southern California, Pasadena, CA USA

**Keywords:** Community mental health, Train-the-trainer, Facilitation, Adaptation, I-PARIHS, Mental illness, Sleep, Circadian, Insomnia, Transdiagnostic, Psychosis, Depression, Anxiety disorder, Bipolar disorder

## Abstract

**Background:**

Grounded in the Integrated Promoting Action on Research Implementation in Health Services (i-PARIHS) framework, we investigated the Train-the-Trainer (TTT) to expand access to evidence-based psychological treatments (EBPTs) in community mental health centers (CMHCs), focusing on the Transdiagnostic Intervention for Sleep and Circadian Dysfunction (TSC).

**Methods:**

Eight Californian counties were cluster-randomized to Standard TSC or an adapted version designed to improve the “fit” of TSC to CMHCs. University-based trainers trained CMHC providers ("Generation 1 providers") in either Adapted or Standard TSC. These trained providers were then trained to become local CMHC trainers (“Generation 1 trainers”), who then trained a new cohort of providers (“Generation 2 providers”) in TSC. Within each county, patients diagnosed with serious mental illness (SMI) were randomized to receive either immediate TSC or usual care and delayed treatment with TSC (UC-DT) from the Generation 2 providers (“Generation 2 patients”). This study focused on 53 Generation 2 providers (Adapted TSC = 47; Standard TSC = 6), and 143 Generation 2 patients (Adapted TSC = 127; Standard TSC = 16) (the larger Adapted sample was driven by recruitment, perhaps reflecting preference for the “fitted” approach). Patient assessments were conducted pre-treatment, post-treatment, and six-month follow-up (6FU). Provider assessments occurred after completing TSC training and post-treatment for each patient treated.

**Results:**

Combining Adapted and Standard, TSC was associated with improvements for Generation 2 patients from pre- to post-treatment in sleep disturbance (*p* < 0.001, *d* = -0.90), sleep-related impairment (*p* = 0.001, *d* = -0.69), psychiatric symptoms (*p* = 0.002, *d* = -0.48), and functional impairment (*p* = 0.002, *d* = -0.54), relative to UC-DT. The effects of sleep disturbance and impairment on the relationship between treatment condition (TSC vs. UC-DT) and psychiatric symptoms and functional impairment were significant. Higher provider perception of TSC fit predicted improvements in selected patient outcomes.

**Conclusion:**

TSC can be delivered by CMHC providers trained by local CMHC trainers with strong outcomes. These data contribute to the dearth of evidence for TTT collected from locally trained providers and from patients treated by local CMHC trainers.

**Trial registration:**

Clinicaltrials.gov identifier: NCT05805657. Registered on March 10, 2023.

**Supplementary Information:**

The online version contains supplementary material available at 10.1186/s13012-025-01467-y.

Contributions to the literature

- Train-the-Trainer holds promise for expanding access to evidence-based treatments
- Using external facilitation grounded in the i-PARIHS framework, external experts trained local CMHC trainers to train other CMHC providers
- CMHC providers trained by the local CMHC trainers effectively delivered a transdiagnostic sleep treatment
- The sleep treatment improved outcomes for CMHC patients with serious mental illness
- Providers rated the sleep treatment as a strong fit for the CMHC context
- Higher provider ratings of fit were associated with better patient outcomes

## Background

According to the Integrated Promoting Action on Research Implementation in Health Services (i-PARIHS) framework [1], the successful implementation of an evidence-based psychological treatment (EBPT) into practice is a function of the quality of evidence for the innovation, the recipients of the innovation, the characteristics of the context into which the innovation will be implemented, and the approach by which the innovation is integrated or facilitated into the context. The present study – focused on the implementation of the Transdiagnostic Intervention for Sleep and Circadian dysfunction (TSC) via Train-the-Trainer (TTT) – will be introduced through the lens of the i-PARIHS framework (also see Table 1).

**Table 1:** Overview of i-PARIHS Core Constructs, Background Considerations, and Key Findings

i-PARIHS core constructs	Background considerations	Key findings from the present study
Innovation		
Efficacy data for TTT	Promising data on TTT for EBPTs but insufficient research, some mixed findings and methodological problems with existing research	TTT is an effective approach to delivering TSC in CMHCs
Consideration of the characteristics of TTT that impact uptake	Community partners saw value in TTT as a low-cost path to implementing TSC and other new EBPTs	TTT can be feasible within CMHCs
Aligning evidence with local priorities and practice	Staff turnover must be considered when planning TTT. TSC training and training to train other providers had potential to create excessive burden on Generation 2 local CMHC trainers and providers	Due to staff turnover, multiple trainers and providers were trained. Local CMHC trainers can train future cohorts of providers and/or new local CMHC trainers as needed in response to staff turnover and patient demand. The dose and timing of training was designed to reduce burden on local CMHC trainers and providers
Efficacy data for TSC	The standard version of TSC had been associated with improvements in outcomes, when delivered by providers employed in an academic setting and CMHC providers trained by expert trainers	TSC alongside usual care is superior to usual care alone. The providers of TSC were employed in CMHC contexts and were trained to deliver TSC by CMHC trainers. An Adapted and Standard version of TSC yielded positive outcomes
Consideration of the characteristics of TSC that impact uptake	While engaging with community partners, there was a clear need and preference for treatments with improved feasibility	The length and complexity of Standard TSC may have contributed to the lower recruitment rates and higher drop-out, compared to Adapted TSC. There were no significant differences between Adapted and Standard TSC on the number of treatment sessions completed. The number of sessions completed for Standard was below the 8 sessions that was recommended. Delivering 8 sessions in the CMHC context may be unrealistic. We provided guidance on how to integrate TSC into sessions alongside other treatments in order to reduce burden and increase feasibility
Aligning evidence with local priorities and practice	Adapted TSC was designed to fit with local needs, including fewer and shorter sessions and trainings	Provider ratings of the fit and credibility of Adapted TSC did not differ from Standard TSC. Generation 2 providers recognized the value and practicality of both Adapted and Standard TranS-C and perceived that TSC was a fit with their expectations and needs within the CMHC setting
Recipient		
People diagnosed with SMI who received TSC	In a prior qualitative research [2], concerns were raised about potential cognitive overload experienced by patients who received the standard version of TSC	Combining Adapted and Standard TSC, patient improvements were observed in sleep, psychiatric symptoms and functional impairment at the post-treatment assessment. Improvements in psychiatric symptoms and functional impairment were mediated through the proposed mechanism of change – namely, sleep and circadian functioning
CMHC providers who were trained by CMHC trainers to deliver TSC	CMHC providers have insufficient time and resources, carry a heavy caseload, and the patients they serve experience high rates of comorbidity and complexity. Training and supervision in EBPTs tend to not be reimbursed by payers. In a prior qualitative research [3], concerns were raised about the fit between the standard version of TSC and the high workload of providers	Providers in both conditions rated TSC as acceptable, appropriate and feasible
Context		
Local level (micro)	The micro level was the main focus of facilitation. The type and intensity of facilitation varied across providers and sites. Example activities: Establishing CE credits for participating in training and to help providers meet license requirements; offering certification in TSC for CMHC providers and trainers; providing leadership and professional development opportunities; facilitating providers to be seen as sleep experts by county leadership and providing networking opportunities through our cross-county meetings	Findings for the present study focused on the innovation and recipient levels
Organizational level (meso)	Example activities: organization-wide trainings; establishing relationships with leadership; email listserve; meetings between leaders at different organizations to solve commonly-faced problems (e.g., insurance codes, provider incentives, etc.); supporting sites in creating dedicated sleep programs	As above
Outer context/Wider health system (macro)	Example activity: efforts to promote sleep health as essential for mental health	As above
Facilitation		
External facilitation, supported by project leadership	Facilitators’ primary activities were (1) recruiting, training, and providing consultation for local CMHC trainers and (2) recruiting and enrolling Generation 2 providers and patients. While local CMHC trainers were heavily involved in increasing provider adoption and utilization of TSC, the facilitators remained in charge of recruiting and enrolling providers and patients through the formal study procedures (e.g., consent, assessments) to reduce burden. Facilitators also held as-needed consultation for TSC providers across generations, offered certification in sleep treatment and sleep training, processed Continuing Education credits, and organized regular meetings with CMHC leadership to provide ongoing support and problem-solve barriers in implementing TSC. After local CMHC trainers held their first training, the facilitation team gradually transferred select responsibilities to them (e.g., presenting to CMHC providers on advanced sleep-related topics; supervising TSC cases on the path to certification)	Facilitation was effective in supporting CMHCs to promote the adoption of TTT and in supporting providers to deliver TSC. Facilitation was well suited to the variety of unique challenges and obstacles faced by trainers and trainees and at each site

i-PARIHS core constructs are derived from Harvey & Kitson’s theoretical papers [1, 4]. Several entries in this table are identical to Table 1 in the Phase 1 report [5] because several findings replicated the Phase 1 results

### Innovations and recipients

EBPTs typically require specialized training for providers. Prior research has established that an effective approach to training providers in EBPTs includes a training workshop utilizing active learning strategies, a provider manual, and ongoing clinical supervision (e.g., [6–10]). However, barriers to the use of these multicomponent training initiatives in routine practice settings include insufficient time and funding, shortage of trainers and supervisors, and staff turnover (e.g., [11, 12]). As a potential solution, the first “innovation” tested in the present study is the Train-the-Trainer (TTT) which involved the external university-based “expert trainers” training an initial cohort of providers in an EBPT. These providers are referred to as “Generation 1.” These providers were offered additional training on how to *train others* in the EBPT and became “local” trainers. These local CMHC trainers then trained the next cohort of providers within their organization, referred to as “Generation 2.”

Although relatively few studies have been conducted on TTT for EBPTs, the existing research has been encouraging [11, 13, 14]. At the provider-level, prior studies show no difference between generations on select outcomes, such as training effectiveness [15], provider competence [16–19] and fidelity [20]. However, there is also evidence of poorer outcomes in Generation 2 relative to expert-led trainings in the domains of provider skill acquisition [16, 21], quality case materials [17], provider knowledge about the EBT [11] and provider satisfaction with the training [11]. Less research has measured TTT patient-level outcomes and relatively few clinical populations and contexts have been investigated [17, 22, 23]. Furthermore, the existing research is qualified by small samples and methodological limitations [7, 8]. The present study aims to help fill these gaps by focusing on two groups of “recipients” (per i-PARIHS). The main focus is on the patient recipients who received the EBPT from Generation 2 providers. This group will be referred to as “Generation 2 patients.” An additional focus is on the Generation 2 providers.

The second “innovation” tested was the Transdiagnostic Intervention for Sleep and Circadian Dysfunction (TSC)^1^ [24]. TSC is an EBPT that aims to improve six dimensions of sleep health [25] and targets sleep and circadian dysfunction, which is a common transdiagnostic contributor to serious mental illness (SMI) [26]. In the present study, the patient recipients were SMI patients who had sleep and/or circadian problems. They were randomized to either Standard or Adapted TSC. Standard TSC was developed within a university setting. As described in the protocol papers [27, 28], Adapted TSC was developed to address the concern that Standard TSC may not “fit” a routine practice setting. Thus, Adapted TSC was customized to fit the context for this study using theory, data, end-user input, consideration of TSC’s theoretical underpinnings and mechanisms of action and treatment strategies that addressed the key mechanisms. Guided by prior research (e.g., [29–31]), we sought to determine if a course of TSC improved sleep and reduced functional impairment and psychiatric symptoms, including suicidal ideation/behaviors and substance use [32, 33].

### Context

The context for this study (as per i-PARIHS) was community mental health centers (CMHCs) which, in the United States, are a large provider of affordable mental health services for people who are low-income and diverse with respect to demographic and clinical presentations. CMHC providers often have insufficient time and resources, carry a heavy caseload, and the patients they serve experience high rates of comorbidity and complexity [34, 35]. It can be difficult for CMHC providers to receive training and supervision in EBPTs [36], because of the cost associated with training and supervision. Before broadly recommending TTT to CMHCs and similar services, TTT must first be tested to assess its effectiveness for training providers in EBPTs within CMHCs.

### Facilitation

Facilitation was chosen as the implementation strategy to support TTT due to its strong evidence base (e.g., [37–39]). In this study, each CMHC received direct support from the lead facilitator, a licensed clinical social worker (ERA) with expertise in community mental health and sleep treatment, along with a team of trained facilitators employed by the research team. The facilitation team was overseen by the Principal Investigator (PI; AGH) and received periodic guidance from a facilitation expert (AMK). Facilitation activities were also informed by the Veterans Affairs facilitation manual [40] and Harvey and Kitson’s [41] Facilitation Guide. Additionally, the lead facilitator (ERA) and postdoctoral scholar (LDS) completed the US Department of Veterans Affairs (VA) Behavioral Health Quality Enhancement Research Initiative (BH QUERI) Implementation Facilitation Training and ERA regularly attended BH QUERI’S monthly drop-in consultation group.

### The present study

This paper describes Phase 2 of a three-part hybrid type 2 effectiveness-implementation study. As described in the protocol paper [27], this study builds upon the infrastructure of Phase 1, the Implementation Phase [5, 28]. During the Implementation Phase, sites were cluster-randomized by county to Adapted or Standard TSC with 1:1 allocation. External expert trainers trained an initial cohort of providers (i.e., Generation 1 providers) in TSC. Then, within each county, patients were randomized to receive immediate TSC or usual care and delayed treatment with TSC (UC-DT) from Generation 1 providers. In Phase 1, TSC (combining Adapted and Standard) was associated with improvement from pre- to post-treatment relative to UC-DT. However, Adapted versus Standard TSC did not differ on provider ratings of fit and better fit did not mediate the relation between TSC condition and patient outcome [5].

The first aim of Phase 2 was to assess the effectiveness of TSC, compared to UC-DT, for Generation 2 patients who were treated by Generation 2 providers. We hypothesized that compared to UC-DT, TSC (combining Adapted and Standard) would be associated with larger reductions in the primary patient outcome of sleep disturbance, and the secondary patient outcomes of sleep-related impairment, sleep health, functional impairment, and psychiatric symptoms. To assess sleep and circadian problems as a mediator of the effects of TSC on patient outcomes, we further hypothesized that TSC’s benefits for functional impairment and psychiatric symptoms would be mediated by improvements in sleep and circadian problems. As explained in more detail in the following paragraph, Aims 2 and 3 differ from those that were pre-specified [27]. The second aim was to assess the effects of TSC treatment condition (Adapted vs. Standard) on primary and secondary outcomes in Generation 2. We hypothesized that Adapted TSC, relative to Standard TSC, would be associated with greater improvements from pre- to post-treatment. The third aim was to examine whether provider ratings of the perceived fit of TSC at post-treatment predicts change in patient outcomes at post-treatment. We hypothesized that greater provider perceived fit of TSC at post-treatment would be associated with improvements in patient outcomes at post-treatment, adjusting for perceived fit at post-training and outcome at pre-treatment. Exploratory analyses focused on (1) comparing Adapted and Standard TSC on patient perceptions of credibility/improvement and select PhenX Toolkit outcomes measuring suicidal ideation/behaviors and substance use; and (2) determining whether treatment effects of TSC versus UC-DT are moderated by risk factors.

Aim 1 and the exploratory analyses were pre-specified in the protocol paper [27]. However, due to the small sample size for provider variables in the Standard condition, Aims 2 and 3 that were pre-specified in the protocol paper could not yield reliable estimates. These results are reported in Additional File 1, see Supplement Tables 1, 2 and 3. The rationale for the new Aims 2 and 3 that are presented above is three-fold. First, these reflect the original intent of the pre-specified aims in that both versions of Aim 2 examine the effects of Adapted vs. Standard TSC on outcomes and both versions of Aim 3 examine perceived fit and outcomes among Gen 2 providers. Second, the new aims?accommodate sample size constraints. Third, in the protocol paper, we state that "If there are too many findings to reasonably interpret in one paper, we may separate some of the findings into two or more papers" (p. 4) [27]. In line with this, Exploratory Aim 1 from the protocol paper warranted a separate report comparing Generations 1 and 2 on patient outcomes [42].

## Method

### Participants

This study focused on 53 Generation 2 providers (Adapted TSC = 47; Standard TSC = 6), and 143 Generation 2 patients (Adapted TSC = 127; Standard TSC = 16). Participants were recruited from CMHCs and consisted of Generation 2 providers who had been trained by local CMHC trainers and Generation 2 patients. Participants were masked to condition (Adapted vs. Standard TSC), but not patient treatment allocation (immediate vs. delayed). All CMHC sites from the Implementation Phase were invited to participate in the TTT Phase. The inclusion criteria for selecting the CMHC sites for the Implementation Phase were: 1) provision of publicly funded adult mental health outpatient services and 2) support from CMHC leadership.

CMHC sites in the following eight counties in California, USA participated: Alameda, Contra Costa, Kings, Monterey, Placer, Santa Cruz, Solano, and Santa Clara. There were 29 CMHC trainers (Adapted TSC = 20; Standard TSC = 9), 53 CMHC providers (Adapted TSC = 47; Standard TSC = 6) and 143 CMHC patients. Of the patients, 78 were randomized to receive TSC immediately (Adapted TSC = 70; Standard TSC = 8) and 65 were randomized to UC-DT (Adapted TSC = 57; Standard TSC = 8). The larger number of providers and patients in Adapted TSC was driven by stronger recruitment in the counties cluster randomized to this condition and perhaps also provider and patient preference for the shorter, “fitted” approach, as compared to Standard TSC.

The inclusion criteria for local CMHC trainers were: 1) employed in participating CMHCs; 2) completed a Generation 1 TSC training (i.e., led by UC Berkeley expert trainers); and 3) volunteered to participate and formally consented to participate.

CMHCs determined eligibility for Generation 2 providers (e.g., case managers, nurses, psychiatrists, training department staff), because this mirrors their real-world practice of determining who acquires additional training. For some CMHCs, this involved mandating TSC training for all untrained staff, whereas in others, leadership advertised the opportunity and allowed anyone who was interested to register. The other inclusion criteria for Generation 2 providers were: 1) employed or able to deliver patient-facing services to patients within the CMHC; 2) interested in learning and delivering TSC; and 3) voluntarily consented to participate.

The inclusion criteria for patients were: 1) aged 18 years and older; 2) met criteria for an SMI per self-report and confirmed by referring provider or administration of the Mini International Neuropsychiatric Interview (DSM-5, Version 7.0.0) by a licensed clinical social worker on the research team; 3) exhibited a sleep or circadian disturbance as determined by endorsing 4 (quite a bit) or 5 (very much), or the equivalent for reverse scored items, on one or more items on the PROMIS-Sleep Disturbance [43, 44]; 4) guaranteed place to sleep for at least two months that is not a shelter; 5) receiving the standard of care for the SMI and consented to regular communications between the research team and provider; and 6) consented to access their medical record and to participate in the study.

Patients were excluded if they met any of the following criteria: 1) presence of an active and progressive physical illness or neurological degenerative disease that was directly related to the onset and course of the sleep and circadian problems, or that made participation in the study unfeasible, as assessed by the Checklist of Medical Conditions and Symptoms on the Duke Structured Interview for Sleep Disorders [45] and clinical interview; 2) presence of substance abuse/dependence only if it made participation in the study unfeasible; 3) current active intent or plan to commit suicide (those with suicidal ideation are eligible) only if it made participation in the study unfeasible, or homicide risk^2^; 4) night shift work for more than two nights per week in the past three months (i.e., regularly scheduled work from 12 a.m. – 6 a.m.); or 5) pregnant or breastfeeding. The rationale for.

### Interventions

Two variations of TSC were tested: Adapted TSC and Standard TSC. Both were delivered alongside the usual care offered by each CMHC. The control condition was usual care followed by delayed treatment (UC-DT). See Additional File 2 for more detailed description of all conditions.

#### Standard TSC

CMHC providers were trained to deliver Standard TSC across eight 50-min, weekly sessions [24]. It consisted of 4 cross-cutting modules featured in every session, 4 core modules, and 7 optional modules, used based on clinical presentation, treatment goals, and provider case conceptualization. Training for the Standard TSC condition consisted of a 1-day workshop (i.e., 6–8 h) or two, 3-h training blocks, based on CMHC preference.

#### Adapted TSC

We grounded the process for adapting TSC in theory, data, and end-user input. Adapted TSC was delivered by CMHC providers across four, 20-min, weekly sessions (see Additional File 2 for description). Treatment consisted of the same four *cross-cutting modules* and three of the four *core modules* as Standard TSC along with one *optional module* focused on reducing sleep-related worry. Training for the Adapted TSC condition consisted of four, 1-h workshops or two, 2-h workshops, based on CMHC preferences.

#### Usual care and delayed treatment with TSC (UC-DT)

In UC-DT, patients began with usual care for four or eight weeks, depending on whether their CMHC was randomized to Adapted TSC or Standard TSC, respectively. After the delay, they received Adapted or Standard TSC, similarly based on the condition to which their CMHC had been randomized. Usual care in CMHCs involves working with a service provider—such as a psychologist, case manager, occupational therapist, psychiatrist, or nurse practitioner—who delivers mental health support within their professional scope.

### Measures

In addition to the measures below, a sociodemographics form was completed by providers and patients. Only measures analyzed for the aims of this paper are briefly reported below. See Additional File 3 for further details on each measure.

#### Generation 2 Patients

##### Sleep disturbance

The 8-item PROMIS-Sleep Disturbance (PROMIS-SD) assessed disruption to sleep (e.g., trouble staying asleep) over the past seven days and has demonstrated acceptable reliability and validity [43, 44]. This was the primary outcome for the patient-level analyses.

##### Sleep-related impairment

The 8-item PROMIS-Sleep Related Impairment (PROMIS-SRI) assessed daytime impairment related to sleep problems using the same scale as the PROMIS-SD.

##### Functional impairment

Functional impairment was assessed via the Sheehan Disability Scale (SDS) [46] which has demonstrated good reliability and validity [46].

##### Overall sleep health

The Sleep Health Composite measured overall sleep health for the complexity of sleep and circadian problems experienced by people diagnosed with SMI and that are covered by TSC [47]. The initial validity of this measure has been established [47].

##### Psychiatric symptoms

The DSM-5 Cross-Cutting Measure assessed psychiatric symptoms across 13 mental health domains (e.g., depression, anger, mania, psychosis, substance use). This measure has demonstrated good test–retest reliability and clinical utility [48, 49].

##### PhenX toolkit

[50]. Two subscales from the screening version of the Columbia-Suicide Severity Rating Scale—Severity of Suicidal Ideation and Suicidal Behavior—were administered. The PhenX ‘Alcohol – 30-Day Quantity and Frequency’, ‘Tobacco – 30 Day Quantity and Frequency’, ‘Substances – 30-Day Frequency’, and ‘Supplemental Beverage Questionnaire’ were used to assess alcohol, tobacco, psychoactive substance, and caffeine consumption over the past 30 days.

##### Credibility and perceived improvement

At the post-treatment assessment, perceptions of TSC’s credibility and symptom improvement were assessed by four questions adapted from the Credibility/Expectancy Questionnaire (CEQ) (Devilly & Borkovec, 2000).

#### Generation 2 Providers

##### Acceptability

Providers rated the acceptability of TSC via the *Acceptability of Intervention Measure* (AIM) (Weiner et al., 2017) which has satisfactory validity, internal reliability, test–retest reliability, and sensitivity to change [51]. This was the primary outcome for the provider-level analyses.

##### Appropriateness and feasibility

Providers rated the appropriateness and feasibility of TSC via the *Feasibility of Intervention Measure* (FIM) and *Intervention Appropriateness Measure* (IAM) [51].

##### Number of TSC sessions

The number of sessions delivered to each enrolled patient by each provider was counted.

##### Occupation

Providers were asked to report their current position, professional degree, and work history, including their caseload, theoretical orientation, licensure status, and previous training in sleep treatment.

### Procedure

CMHCs and patients were randomized through a computerized randomization sequence. We did not stratify during randomization at the CMHC level. When randomizing patients, we stratified for the presence of psychosis or not (current), presence of substance use or not (current) and age (≥ 50 or not), as there is evidence these variables can impact sleep or treatment outcome [52–54]. Only the facilitators, assessors, and research team (i.e., not CMHCs, local trainers, providers, or patients) were privy to which CMHCs and patients were allocated to which TSC treatment condition (Adapted versus Standard). CMHC providers, local CMHC trainers, and patients knew whether their patients had been randomized to receive the immediate or delayed treatment. A facilitator informed the local trainer once a patient could start having sessions, who then informed the provider. In the immediate condition, the provider is asked to begin sessions as soon as possible. In the delayed condition, the provider was asked to wait until after the patient had completed the post-delay assessment (i.e., approximately four weeks in the Adapted condition or eight weeks in the Standard condition).

Generation 2 provider and patient assessments were conducted by experienced assessors who also handled the consent process to reduce participant burden. As they needed to share study details (e.g., number of assessments, treatment sessions), assessors were unmasked at pre-treatment. Efforts were made to keep assessors masked at post-treatment and 6FU. Assessors received thorough training and ongoing supervision to ensure survey integrity and minimize bias.

The UC Berkeley facilitation team transitioned CMHC sites from the Implementation Phase to the TTT Phase on a rolling basis. Each site’s readiness for TTT was assessed by the level of provider engagement, the number of patients who had completed sleep treatment, and the supportiveness of leadership. The first site was transitioned to TTT in December 2020, and all sites were transitioned by December 2022. Facilitator’s primary activities in the TTT Phase are summarized in Table 1.

Local CMHC trainers led Generation 2 trainings independent of the expert trainer. Due to the COVID-19 pandemic and in accordance with local preferences and requirements, all Generation 2 trainings were delivered over Zoom. Generation 2 trainers had varying degrees of access to and familiarity with Zoom and little time to master it. Thus, for the first training led by each local trainer, a UC Berkeley facilitator attended the meeting to provide support with Zoom technology. The facilitator only answered content-related questions if requested by the local trainer. The UC Berkeley facilitator had some content-knowledge regarding TSC, but they were not trainers. After the first training, facilitator attendance was offered but not required.

Following conducting their first training, local CMHC trainers began holding drop-in supervision hours for Generation 2 providers. The expert trainer continued to hold drop-in consultation hours, open to Generation 1 providers. Also, the expert trainer held individual consultation for the local CMHC trainers to support their transition to a supervision role. Overall, we viewed the supports detailed above to be deviations from the ideal TTT structure yet crucial within the CMHC context and particularly during the pandemic.

#### Local CMHC trainers

Trainers did not complete assessment batteries for the TTT Phase and are not a focus of this report.

#### Generation 2 Providers

Provider assessments were completed after the provider completed TSC training (i.e., post-training), as well as at post-treatment for each patient they treated.

#### Generation 2 Patients

Patient assessments in the immediate TSC treatment conditions were completed at pre-treatment, post-treatment, and six months after treatment (6FU). Patient assessments in the UC-DT condition were completed at pre-treatment and four or eight weeks after pre-treatment (i.e., at the end of usual care and before delayed treatment with TSC referred to as post UC-DT), depending on whether their county has been randomized to Adapted or Standard TSC, respectively. Patients did not complete a 6FU assessment after the delay portion of the UC-DT. This was a compromise made with CMHC partners, so that patients would have minimal wait time before receiving treatment. As a result, patients started delayed treatment with TSC after the post UC-DT assessment. Following delayed TSC treatment, patients completed the same assessments as those in the immediate TSC condition i.e., post-treatment and 6FU.

### Trial registration, data transparency and openness

All research materials, data, and analysis code are available from the authors upon request. This study was preregistered on clinicaltrials.gov (identifier: NCT05805657), a protocol paper was published [27] and the study received approval from the Committee for the Protection of Human Subjects at the University of California, Berkeley. Raw data for most outcomes reported here have been uploaded into the National Data Archive. An update was made to clinicaltrials.gov to clarify that, for the primary outcome measures, assessments at mid-treatment were not of primary interest. This error was rectified by moving the mid-treatment assessment to “Other outcome measures”.

#### Analyses

Analyses were conducted with Stata Version 16.1. Percent of missing data for each aim are presented in Supplement Tables 4–7, Additional File 1.

##### Multilevel models (Aims 1 & 2 and Exploratory Aims 1 & 2)

Multilevel models (MLMs) were used to account for multiple observations nested within patient [55]. All MLMs compared pre-treatment to post-treatment and, for level 1, included a dummy-coded time indicator as the predictor (1 = post-treatment, pre-treatment as the reference). Exploratory Aim 1 also compared pre-treatment to 6FU follow-up and included an additional time indicator accordingly. For all MLMs, the level 2 equation included dummy-coded treatment condition (*Aim 1 and Exploratory Aim 2:* 1 = immediate TSC, with UC-DT as the reference; *Aim 2 and Exploratory Aim 1:* 1 = Adapted TSC, with Standard as reference) and treatment-by-time interaction terms, which were the parameters of interest. Additionally, Exploratory Aim 2 included three-way interactions between time, treatment, and the following pre-specified moderators: sex (dummy coded: 0 = male, 1 = female), age (dummy coded: 0 = < 50, 1 = ≥ 50), and continuous baseline variables of PROMIS-SD, PROMIS-SRI, SDS, and DSM-5 Cross-Cutting. Significant interactions were interpreted using graphs.

We list the outcomes included in each MLM. For Aim 1 and 2 MLMs, the outcomes were PROMIS-SD, PROMIS-SRI, Sleep Health Composite, DSM-5 Cross-Cutting, and SDS. For Exploratory Aim 1, the MLM outcomes were severity of suicidal ideation, average number of caffeinated drinks per day, and number of days the patient consumed alcohol in the past 30 days. For Exploratory Aim 2, the outcomes mirrored Aims 1 and 2. Most outcomes were continuous, except for the following binary outcomes tested in Exploratory Aim 1: suicidal thoughts and behaviors and substance use. For these outcomes, multilevel logistic regression was used. However, because few participants endorsed these items, the models would not converge. Instead, the frequencies of patients’ endorsement of each item are presented in Additional File 1, Supplement Table 8.

##### Linear regression models (Aim 3 and Exploratory Aim 1)

For Aim 3, residualized change models [56] were conducted via multiple linear regression to evaluate whether perceived fit at post-treatment predicted patient outcomes at post-treatment, adjusting for pre-treatment levels. The predictor was AIM, FIM, or IAM at post-treatment, and the outcomes were PROMIS-SD, PROMIS-SRI, Sleep Health Composite, DSM-5 Cross-Cutting, and SDS at post-treatment.

For Exploratory Aim 1, linear regression models were used to test the effects of TSC treatment condition on credibility and perceived improvement at post-treatment. The predictor was dummy-coded TSC treatment condition (1 = Adapted TSC, with Standard as reference) and the outcomes were credibility, expectancy, and total CEQ.

##### Structural Equation Modeling (SEM) (Aim 1)

For the mediation models in Aim 1, SEM was used. The predictor was condition (immediate TSC vs. UC-DT), the mediator was PROMIS-SD or PROMIS-SRI at post-treatment, and the outcomes were DSM-5 Cross-Cutting and SDS at post-treatment. For all SEMs, the parameter of interest was the indirect effect. Of note, statisticians and methodologists have argued that contemporaneous models, whereby the mediators and outcomes are both measured at post-treatment, may confer advantages for clinical trials, because these timepoints capture the interval during which the greatest changes are most likely to occur in the mediators and outcomes (e.g., [57–59]).

## Results

**Fig. 1:**
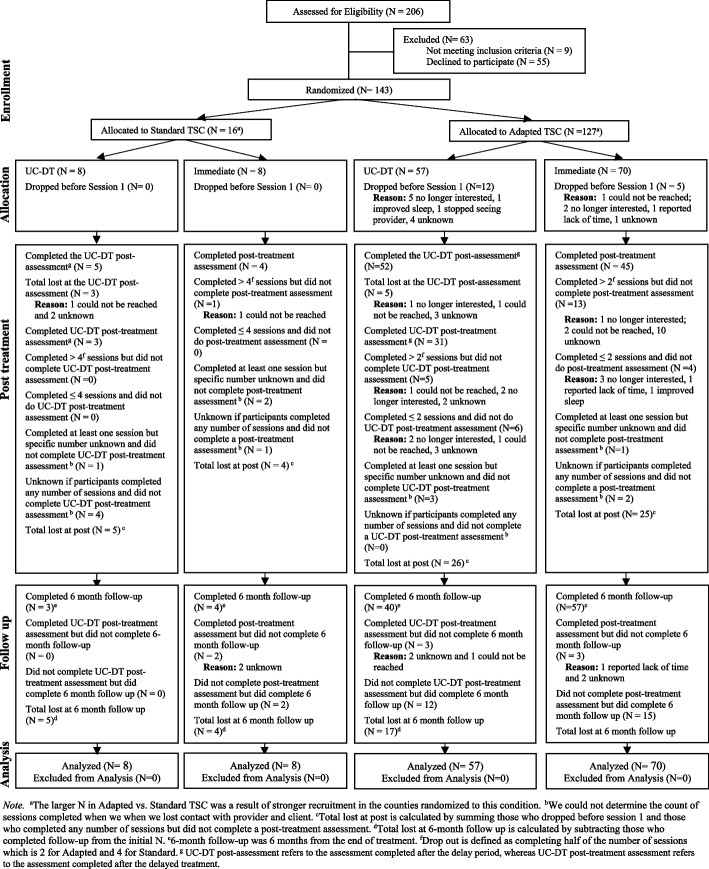
CONSORT Diagram Illustrating the Flow of Patients Through the Study. Note. ^a^The larger N in Adapted vs. Standard TSC was a result of stronger recruitment in the counties randomized to this condition. ^b^We could not determine the count of sessions completed when we when we lost contact with provider and client. ^c^Total lost at post is calculated by summing those who dropped before session 1 and those who completed any number of sessions but did not complete a post-treatment assessment. ^d^Total lost at 6-month follow up is calculated by subtracting those who completed follow-up from the initial N. ^e^6-month follow-up was 6 months from the end of treatment. ^f^Drop out is defined as completing half of the number of sessions which is 2 for Adapted and 4 for Standard. ^g^ UC-DT post-assessment refers to the assessment completed after the delay period, whereas UC-DT post-treatment assessment refers to the assessment completed after the delayed treatment

**Fig. 2:**
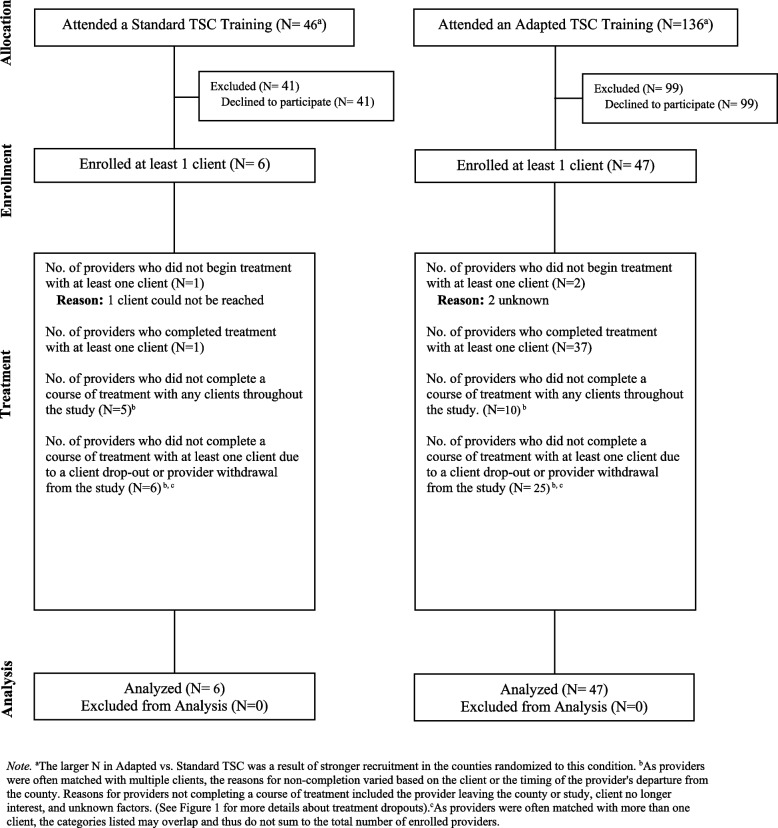
CONSORT Diagram Illustrating the Flow of Providers Through the Study. Note. ^a^The larger N in Adapted vs. Standard TSC was a result of stronger recruitment in the counties randomized to this condition. ^b^As providers were often matched with multiple clients, the reasons for non-completion varied based on the client or the timing of the provider's departure from the county. Reasons for providers not completing a course of treatment included the provider leaving the county or study, client no longer interest, and unknown factors. (See Fig. 1 for more details about treatment dropouts). ^c^As providers were often matched with more than one client, the categories listed may overlap and thus do not sum to the total number of enrolled providers

See Fig. 1 for the CONSORT diagram for patients. Attrition rates were significantly higher in Standard than Adapted TSC during the treatment phase (56.3% in Standard; 26.8% in Adapted; *χ*^*2*^ = 4.55, df = 1, *p* = 0.03), but not significantly different prior to Session 1 (0% in Standard; 13.4% in Adapted; *χ*^*2*^ = 1.32, df = 1, *p* = 0.25), or at 6FU (12.5% in Standard; 4.7% in Adapted; *χ*^*2*^ = 0.49, df = 1, *p* = 0.49). See Fig. 2 for the CONSORT diagram for providers. Patient and provider demographic variables at pre-treatment by TSC condition (Adapted vs. Standard) are presented in Tables 2 and 3, respectively. Further information on patient and provider differences by TSC condition are reported in Additional File 1. Additional File 1, Supplement Table 9 presents the patient demographics by immediate TSC vs. UC-DT condition.

**Table 2:** Patient demographics and number of sessions by treatment condition (Standard versus Adapted TSC) at Pre-Treatment collapsed over UC-DT and immediate TSC conditions

**Characteristic**	**Standard TSC (*n* = 16)**		**Adapted TSC (*n* = 127)**			
	***n***	***%***	***n***	***%***	**χ**^**2**^	***p*** **-value**
Sex					0.01	0.75
Female	9	56.25	81	63.78		
Male	7	43.75	46	36.22		
Ethnicity					0.66	0.42
Hispanic or Latino	5	31.25	26	20.47		
Not Hispanic or Latino	10	62.50	101	79.53		
Race					12.70	0.05
American Indian/Alaska Native	0	0.00	7	5.51		
Native Hawaiian/Pacific Islander	0	0.00	3	2.36		
Asian	2	12.50	13	10.24		
Black or African American	5	31.25	24	18.90		
White	4	25.00	54	42.52		
More than one race	1	6.25	20	15.75		
Other/category not listed	4	25.00	6	4.72		
Education					7.87	0.10
High school graduate or below	3	18.75	11	8.66		
Some or completed college or vocational school	12	75.00	79	64.20		
Some or completed graduate school	4	25.00	34	26.77		
Other/category not listed	1	6.25	0	0.00		
Missing/declined to answer	0	0.00	3	2.36		
Employment					3.34	0.34
Full-time	4	25.00	24	18.90		
Part-time	3	18.75	19	14.96		
Not employed	7	43.75	79	62.20		
Other/category not listed	2	12.50	5	3.94		
Civil Status					8.11	0.04
Partnered	3	25.00	25	19.69		
Unpartnered	12	75.00	101	79.53		
Other/category not listed	1	6.25	0	0.00		
Missing/declined to answer	0	0.00	1	0.79		
Living Arrangement					4.38	0.36
Alone	1	6.25	25	19.69		
With family	12	75.00	69	54.33		
With friend or roommate or pet	2	12.50	20	15.75		
Supported housing	0	0.00	10	7.87		
Other/category not listed	0	0.00	3	2.36		
Government Assistance^a^					4.01	0.86
Unemployment	0	0.00	3	2.36		
Medicare	1	6.25	11	8.66		
Medicaid	4	25.00	45	35.43		
Social Security	3	18.75	13	10.24		
Food Stamps	3	18.75	27	21.26		
SSI/SSDI	2	12.50	25	19.69		
SNAP	1	6.25	14	11.02		
None	0	0.00	0	0.00		
Other/category not listed	4	25.00	17	13.39		
Missing/declined to answer	5	31.25	38	29.92		
Annual Personal Income					11.48	0.12
< $10,000	1	6.25	38	29.92		
$10,000-$20,000	6	37.50	28	22.05		
$20,000-$30,000	3	18.75	7	5.51		
$30,00-$40,000	0	0.00	10	17.87		
$40,000-$50,000	2	12.50	6	4.72		
> = $50,000	1	6.25	17	13.39		
I don’t know my income	3	18.75	20	15.75		
Missing/declined to answer	0	0.00	1	0.79		
Annual Household income					14.09	0.05
< $10,000	1	6.25	20	15.75		
$10,000-$20,000	4	25.00	23	18.11		
$20,000-$30,000	5	31.25	10	7.87		
$30,00-$40,000	0	0.00	7	5.51		
$40,000-$50,000	2	12.50	5	3.94		
> = $50,000	1	6.25	31	24.41		
I don’t know my income	3	18.75	29	22.83		
Missing/declined to answer	0	0.00	2	1.57		
Self-reported diagnosis^b^					14.76	0.14
Neurodevelopmental disorders	1	6.25	21	16.54		
Psychosis	3	18.75	33	25.98		
Mood Disorder Features (Bipolar)	2	12.50	23	18.11		
Mood Disorder Features (Unipolar)	7	43.75	59	46.46		
Anxiety disorders	8	50.00	64	50.39		
Obsessive–compulsive and related disorders	0	0.00	5	3.94		
Trauma and stressor-related disorders	2	12.50	31	24.41		
Dissociative disorders	0	0.00	0	0.00		
Personality disorders	0	0.00	2	1.57		
Feeding and eating disorders	0	0.00	0	0.00		
Substance-related and addictive disorders	0	0.00	5	3.94		
Other/category not listed	2	12.50	1	0.79		
Missing/declined to answer	1	6.25	14	11.02		
	***Mean***	***SD***	***Mean***	***SD***	***t***	***p*** **-value**
Age	45.00	10.68	43.33	14.08	0.56	0.58
Education (years)	15.00	4.19	14.82	3.57	0.16	0.88
No. of sessions received (all)^c^	3.69	3.32	4.33	3.95	−0.71	0.48
No. of sessions received (completers)^d^	6.33	2.42	5.34	3.70	0.93	0.38

Chi-squared was used for categorical variables, and *t* tests were used for continuous variables

^a^Some patients endorsed more than one government assistance category

^b^Comorbidity was common

^c^Number of TSC sessions received by all enrolled patients in the study

^d^Number of TSC sessions received by patients who completed treatment

**Table 3:** Provider demographics by TSC treatment condition (Standard versus Adapted TSC) at post-training

**Characteristic**	**Standard TSC (*n* = 6)**		**Adapted TSC (*n* = 47)**			
	***n***	***%***	***n***	***%***	**χ**^**2**^	***p*** **-value**
Sex					0.55	0.76
Female	5	83.33	33	70.21		
Male	0	0.00	2	4.26		
Missing/declined to answer	1	16.67	12	25.53		
Ethnicity					0.50	0.78
Hispanic or Latino	1	16.67	8	17.02		
Not Hispanic or Latino	4	66.67	25	53.19		
Missing/declined to answer	1	16.67	14	29.79		
Race					13.80	0.008
Asian	1	16.67	7	14.89		
Black or African American	2	33.33	1	2.13		
White	1	16.67	24	51.06		
More than one race	1	16.67	1	2.13		
Missing/declined to answer	1	16.67	14	29.79		
Degree Type^a^					4.58	0.60
Marriage and Family Therapy	0	0.00	4	8.51		
Psychology	1	16.67	4	8.51		
Social Work	4	66.67	15	31.91		
Nursing	0	0.00	8	17.02		
Medical	0	0.00	1	2.13		
Other	0	0.00	4	8.51		
Missing	1	16.67	12	25.53		
Therapeutic Approach^a^					2.19	0.90
Client Centered	5	83.33	29	61.70		
Family Systems	0	0.00	8	17.02		
CBT	2	33.33	20	42.55		
Psychodynamic	1	16.67	11	23.40		
Humanistic	1	16.67	7	14.89		
Integrative/Holistic	0	0.00	2	4.26		
Missing/declined to answer	1	16.67	13	27.66		
Licensure					0.39	0.82
Licensed	3	50.00	24	51.06		
Not Licensed	2	33.33	11	24.40		
Missing/declined to answer	1	16.67	12	25.53		
	***Mean***	***SD***	***Mean***	***SD***	***t***	***p*** **-value**
Age	39.50	12.40	40.21	10.19	−0.11	0.92
Caseload	33.00	37.24	33.29	40.18	−0.01	0.99
Employment Duration	1.20	1.10	5.41	6.23	−3.54	0.001
Years Since Degree Earned	6.40	6.43	9.63	9.69	−0.98	0.36

Chi-squared was used for categorical variables, and *t* tests were used for continuous variables

*CBT* cognitive behavioral therapy. Caseload = number of patients on caseload. Employment duration = length of time employed at current CMHC in years

^a^Some providers endorsed more than one degree type and therapeutic approach

### Aim 1

See Tables 4 and 5. TSC, relative to UC-DT, was associated with significant improvements from pre- to post-treatment in sleep disturbance, sleep-related impairment, psychiatric symptoms, and overall functional impairment. TSC, relative to UC-DT, was marginal (*p* = 0.09) for the sleep health composite. Sleep disturbance (primary outcome) withstood the Benjamini–Hochberg correction.

**Table 4:** Means, standard deviations, and effect sizes for primary and secondary outcomes

	**Pre-Treatment (for patients) & Post-training (for providers)**				**Post-treatment**				
Patient Outcomes									
	**UC-DT (** ***n*** ** = 65)**		**TSC (** ***n*** ** = 78)**		**UC-DT**		**TSC**		***d***
	**Mean**	**SD**	**Mean**	**SD**	**Mean**	**SD**	**Mean**	**SD**	
PROMIS-SD*	62.05	7.14	62.71	7.69	61.08	8.59	54.74	11.04	−0.90
PROMIS-SRI	59.98	8.79	60.59	8.76	57.88	8.39	52.46	11.9	−0.69
SHC	2.43	1.46	2.31	1.28	2.69	1.44	3.09	1.63	0.43
DSM-5	22.69	9.15	23.68	8.69	20.67	8.46	17.59	9.64	−0.48
SDS	11.86	7.38	12.77	7.62	11.07	7.46	7.86	6.95	−0.54
	**Standard (** ***n*** ** = 16)**		**Adapted (** ***n*** ** = 127)**		**Standard**		**Adapted**		***d***
	**Mean**	**SD**	**Mean**	**SD**	**Mean**	**SD**	**Mean**	**SD**	
PROMIS-SD*	63.81	6.66	62.23	7.52	54.21	12.22	55.21	11.18	0.51
PROMIS-SRI	61.18	10.54	60.2	8.54	52.74	10.56	53.54	11.14	0.02
SHC	2.62	1.33	2.33	1.37	2.50	2.07	3.07	1.52	0.63
DSM-5	22.06	7.84	23.37	9.03	16.14	8.07	18.00	9.34	0.16
SDS	13.50	8.43	12.21	7.40	8.86	7.73	8.36	6.47	0.03
Provider Outcomes									
	**Standard (** ***n*** ** = 16)**		**Adapted (** ***n*** ** = 127)**		**Standard**		**Adapted**		***d***
	**Mean**	**SD**	**Mean**	**SD**	**Mean**	**SD**	**Mean**	**SD**	
AIM	4.9	0.22	4.74	0.41	5	0	4.54	0.58	−0.94
FIM	4.95	0.11	4.59	0.57	4.44	0.72	4.43	0.65	4.36
IAM	5	0	4.79	0.35	4.75	0.5	4.39	0.67	NA

*PROMIS-SD* PROMIS Sleep Disruption, *PROMIS-SD* PROMIS Sleep Disturbance, *PROMIS-SRI* PROMIS Sleep-Related Impairment, *SHC* Sleep Health Composite (note, scored such that higher scores indicate better sleep health), *DSM-5* DSM-5 Cross-Cutting, *SDS* Sheehan Disability Scale, *AIM* Acceptability of Intervention Measure, *FIM* Feasibility of Intervention Measure, *IAM* Intervention Appropriateness measure, *TSC* Transdiagnostic Intervention for Sleep and Circadian Dysfunction, *UC-DT* usual care followed by delayed treatment with TSC, *NA* not applicable (there was no variability in IAM across conditions). Effect sizes are represented with ‘*d*’ and were calculated following Feingold (2009, Eq. 5), using unadjusted change scores (mean difference between pre- and post-treatment) and raw standard deviations at pre-treatment from each treatment condition. The pre-treatment *Ns* reflect the size of the intent-to-treat sample. Please see Supplement Tables 4–7f or missing data by aim, timepoint, and outcome

^*^indicates primary outcome

**Table 5:** Aim 1: Multilevel Modeling Results for Treatment Condition (UC-DT versus TSC) on Patient Outcomes from Pre- to Post-Treatment

	***b***	**SE**	***p-*** **value**
Outcome			
PROMIS-SD	−7.16	1.68	**< 0.001**
PROMIS-SRI	−6.44	2.00	**0.001**
SHC	0.55	0.32	0.09
DSM-5	−4.25	1.38	**0.002**
SDS	−4.33	1.39	**0.002**

Bold indicates significant *p*-values

*b* time-by-treatment interaction, *SE* robust standard errors, *PROMIS-SD* PROMIS Sleep Disturbance, *PROMIS-SRI* PROMIS Sleep-Related Impairment, *SHC* Sleep Health Composite, *DSM-5* DSM-5 Cross-Cutting, *SDS* Sheehan Disability Scale

See Table 6 for SEM results. The indirect effects of treatment condition (TSC vs. UCT-DT) on psychiatric symptoms and overall functional impairment via sleep disturbance and sleep-related impairment were significant.

**Table 6:** Aim 1: Mediation Models of Sleep Outcomes on Relations between Treatment Condition (TSC vs. UC-DT) and Psychiatric Symptoms and Overall Functional Impairment at Post-Treatment

	Coefficient	SE	z	*p*	95% Confidence Interval of effect	%MP
Aim 1 Model 1: TSC vs. UC-DT, PROMIS-SD at Post, DSM-5 at Post						
Path a	−6.65	1.77	−3.76	< 0.001	−10.11 −3.19	-
Path b	0.29	0.08	3.73	< 0.001	0.14, 0.45	-
Total effect	−4.26	1.42	−3.00	0.003	−7.04, −1.48	-
Indirect effect	−1.95	0.81	−2.40	0.02	−3.54, −0.36	45.77%
Aim 1 Model 2: TSC vs. UC-DT, PROMIS-SD at Post, SDS at Post						
Path a	−6.50	1.77	−3.68	< 0.001	−9.97, −3.04	-
Path b	0.34	0.08	4.33	< 0.001	0.18, 0.49	-
Total effect	−3.95	1.18	−3.35	0.001	−6.26, −1.64	-
Indirect effect	−2.20	0.70	−3.15	0.002	−3.57, −0.83	55.70%
Aim 1 Model 3: TSC vs. UC-DT, PROMIS-SRI at Post, DSM-5 at Post						
Path a	−6.42	1.92	−3.35	0.001	−10.19, −2.66	-
Path b	0.25	0.06	4.11	< 0.001	0.13, 0.37	-
Total effect	−4.27	1.44	−2.97	0.003	−7.09, −1.45	-
Indirect effect	−1.62	0.63	−2.56	0.01	−2.87, −0.38	37.94%
Aim 1 Model 4: TSC vs. UC-DT, PROMIS-SRI at Post, SDS at Post						
Path a	−6.31	1.89	−3.34	0.001	−10.01, −2.61	-
Path b	0.44	0.06	7.62	< 0.001	0.32, 0.55	-
Total effect	−3.86	1.18	−3.26	0.001	−6.17, −1.54	-
Indirect effect	−2.76	0.86	−3.22	0.001	−4.44, −1.08	71.50%

Significant effects for parameters of primary interest (i.e., indirect effects) are highlighted in bold. "-" indicates that value is not relevant to model. SE = robust standard errors

*%MP* mediated proportion (i.e., the proportion of the total effect that is explained by the indirect effect expressed as a percentage), *TSC* Transdiagnostic Intervention for Sleep and Circadian Dysfunction, *UC-DT* usual care followed by delayed treatment with TSC, *PROMIS-SD* PROMIS Sleep Disturbance, *PROMIS-SRI* PROMIS Sleep-Related Impairment, *SDS* Sheehan Disability Scale, *DSM-5* DSM-5 Cross-Cutting, *POST* post-treatment assessment, *Path a* path from the independent variable to mediator (i.e., Treatment condition PROMIS-SD or PROMIS-SRI), *Path b* path from the mediator to the outcome (PROMIS-SD or PROMIS-SRI DSM-5 Cross Cutting or SDS)

All models adjusted for pre-treatment levels of the relevant mediator (i.e., PROMIS-SD or PROMIS-SRI) and relevant outcome (i.e., DSM-5 Cross-Cutting or SDS)

### Aim 2

See Tables 4 and 7. There were no significant differences between Adapted and Standard TSC on changes from pre- to post-treatment on primary or secondary outcomes.

**Table 7:** Aim 2: Multilevel Modeling Results for TSC Treatment Condition (Standard vs. Adapted) on Patient Outcomes from Pre- to Post-Treatment

	***b***	**SE**	***p-*** **value**
Outcome			
PROMIS-SD	2.22	3.40	0.52
PROMIS-SRI	1.36	3.72	0.72
SHC	1.05	0.66	0.11
DSM-5	1.79	3.05	0.56
SDS	0.84	2.93	0.78

*b* time-by-treatment interaction, *SE* standard errors, *PROMIS-SD* PROMIS Sleep Disturbance, *PROMIS-SRI* PROMIS Sleep-Related Impairment, *SHC* Sleep Health Composite, *DSM-5* DSM-5 Cross-Cutting, *SDS* Sheehan Disability Scale

### Aim 3

See Tables 4 and 8. Greater provider perceptions of acceptability (AIM) predicted improvements in patient sleep-related impairment at post-treatment. Greater feasibility (FIM) predicted improvements in patient sleep-related impairment and psychiatric symptoms at post-treatment. Greater FIM also marginally predicted improvements in sleep health composite and functional impairment. Greater appropriateness (IAM) predicted improvements in patient sleep-related impairment and psychiatric symptoms. Provider ratings on the AIM, FIM and IAM ranged from 4.39 to 5 on the 1 (completely disagree) to 5 (completely agree) scale.

**Table 8:** Aim 3: Provider perceived fit predicting patient outcomes

	*b*	SE	*p-*value	*n*_*p*_^2^
Predictor: AIM				
PROMIS-SD	−10.89	4.31	0.02	0.12^D^
PROMIS-SRI	−3.90	3.69	0.30	0.02
SHC	0.45	0.84	0.59	0.01
DSM-5	−3.46	3.61	0.34	0.03
SDS	−1.41	2.04	0.49	0.01
Predictor: FIM				
PROMIS-SD	−10.37	2.86	0.001	0.21
PROMIS-SRI	−4.45	3.06	0.15	0.04
SHC	0.89	0.46	0.06	0.07^D^
DSM-5	−5.65	2.16	0.01	0.12^D^
SDS	−2.07	1.21	0.09	0.04^D^
Predictor: IAM				
PROMIS-SD	−6.26	2.95	0.04	0.09^D^
PROMIS-SRI	0.79	2.81	0.78	0.002
SHC	0.72	0.47	0.14	0.05
DSM-5	−5.54	1.98	0.01	0.14
SDS	−1.62	1.44	0.27	0.03

*b* effect of fit at post-treatment on patient outcomes, *SE* robust standard errors, *n*_*p*_^2^ partial eta squared, *AIM* Acceptability of Intervention Measure, *FIM* Feasibility of Intervention Measure, *IAM* Intervention Appropriateness measure, *PROMIS-SD* PROMIS Sleep Disruption, *PROMIS-SD* PROMIS Sleep Disturbance, *PROMIS-SRI* PROMIS Sleep-Related Impairment, *SHC* Sleep Health Composite (note, scored such that higher scores indicate better sleep health), *DSM-5* DSM-5 Cross-Cutting, *SDS* Sheehan Disability Scale, ^D^ Differences when including TSC condition (Standard vs. Adapted) and provider degree as covariates instead of county, all comparisons become less significant. AIM predicting sleep disturbance changes from significant to non-significant (*b* = −5.74, *SE* = 4.31, *p* = 0.19, *n*_*p*_^2^ = 0.04). FIM predicting sleep health composite (*b* = 0.64, *SE* = 0.50, *p* = 0.21, *n*_*p*_^2^ = 0.04) and functional impairment (*b* = −1.33, *SE* = 1.39, *p* = 0.35, *n*_*p*_^2^ = 0.02) changes from marginally significant to non-significant. FIM predicting psychiatric symptoms changes from significant to marginally significant (*b* = −4.12, *SE* = 2.31, *p* = 0.08, *n*_*p*_^2^ = 0.07). IAM predicting sleep disturbance changes from significant to marginally significant (*b* = −4.93, *SE* = 2.84, *p* = 0.09, *n*_*p*_^2^ = 0.06)

### Exploratory aims

See Additional File 1, Supplement Tables 10 and 11. For Exploratory Aim 1, there were no significant differences between Adapted and Standard TSC on suicidal ideation severity, average daily caffeine use, or past 30-day alcohol use (all *p*s > 0.10).

There were no differences between Adapted versus Standard TSC on credibility, perceived improvement, or total CEQ (all *p*s > 0.10). At post-treatment, the mean of the credibility items was 7.39 (*SD* = 1.45) on the 0 (not at all) to 9 (very) scale and mean perceived improvement was 56.13% (*SD* = 30.63).

For Exploratory Aim 2, see Additional File 1, Supplement Table 12. Baseline sleep related-impairment marginally moderated the effects of treatment (UC-DT versus immediate TSC) on functional impairment from pre- to post-treatment. This effect was such that at the lowest level of baseline sleep-related impairment there was no difference between UC-DT vs. immediate TSC conditions (*p* = 0.63), but at the highest level of baseline sleep-related impairment there were greater improvements in functional impairment for the immediate relative to the delayed group (*p* = 0.01). None of the other planned demographics or baseline clinical symptoms moderated the effects of treatment on patient outcomes from pre- to post-treatment (all *p*s > 0.10).

## Discussion

We sought to determine if TTT is an effective approach to delivering TSC in CMHCs. Table 1 and this discussion frames the findings through i-PARIHS. There are several overarching issues that emerged with regard to the recipients. The patient sample was diverse, largely unpartnered, low income, unemployed, and living with family. Most providers were female social workers using client-centered approaches, licensed, and managing high caseloads (avg. 33 patients). Providers rated both Adapted and Standard TSC as highly appropriate, acceptable, and feasible which suggests that Generation 2 providers recognized the value and practicality of both approaches. Although Standard providers were trained for 8 sessions and Adapted for 4, no significant difference was found in sessions completed. This may reflect feasibility issues in CMHCs and the higher attrition in the Standard group.

We first focus on outcomes for the patient-level recipients of the innovations delivered in this study. Consistent with our hypothesis, Generation 2 patients treated with TSC reported larger reductions in sleep disturbance, sleep-related impairment, functional impairment, and psychiatric symptoms, relative to UC-DT. The same pattern of findings was evident for the sleep health composite, except the difference between TSC and UC-DT was marginally significant. These findings are important for at least five reasons. First, they extend prior research conducted with university-based providers [60] and CMHC providers trained by university-based trainers [5] and add to the growing support for both TSC [61–64] and the Sleep Health Framework [25, 47]. Second, they add to the evidence for TTT and to the handful of reports on Generation 2 patient-level outcomes from TTT [17, 22, 23]. Third, they demonstrate the feasibility and effectiveness of TSC and TTT in CMHCs, with potential for broader application in under-resourced settings. Fourth, these results add to the evidence for external facilitation as a successful implementation strategy (e.g., [37–39]) and expand knowledge by demonstrating the success of external facilitators in establishing a TTT structure. Fifth, these findings are particularly encouraging given that the study was conducted during the height of the COVID-19 pandemic, a period known for its well-documented disruptions to sleep and circadian functioning [65].

There was support for the hypothesis that TSC’s benefits for functional impairment and psychiatric symptoms would be mediated by improvements in sleep, as assessed by the PROMIS-SD and PROMIS-SRI. This replicates and extends the parallel finding for the Implementation Phase of this study in which UC Berkeley experts served as the trainers [5] as well as prior research showing that sleep treatment improves symptoms of comorbid mental health conditions (e.g., [29–31]). Within the i-PARIHS framework, these results suggest that facilitation was effective in supporting trainers to train CMHC providers to deliver the innovation (TSC) within the CMHC context, resulting in improved outcomes for the SMI recipients.

For the second aim, contrary to the hypothesis, there were no significant differences between Adapted and Standard TSC. This result aligns with the parallel findings from the Implementation Phase and might be explained by the relative advantages of each approach (see [5]). Of note, Adapted and Standard TSC differed in the number of trainers (Adapted TSC = 20; Standard TSC = 9) and trainees (Adapted TSC = 47; Standard TSC = 6). Further, there was more attrition among patients who participated in Standard (56%) than Adapted (27%). The differential attrition may have reduced our statistical power to detect differences between Adapted and Standard TSC.

For the third aim, we focused on the Generation 2 provider recipients who were trained to deliver TSC via TTT. The hypothesis tested was that greater provider perceived fit at post-treatment would be associated with improvements in patient outcomes at post-treatment. Most of the results were in the predicted direction (see Table 8) with several comparisons reaching statistical significance, and 14 of the 15 comparisons showing small to large effect sizes. Together, these findings add to the growing evidence that underscores the importance of the fit between the treatment and the context [66–68] and extends these findings by showing the importance of fit for better patient outcomes. Consistent with i-PARIHS, these results suggest that the use of facilitation, as well as TTT, was effective in supporting the second group of recipients in the study—CMHC providers—to deliver the innovation (TSC) despite the many challenges faced at the local, organizational and outer context of CMHCs (see Table 1 for details).

The only significant moderator of treatment effects was that TSC’s effect (compared to UC-DT) on improving functional impairment was particularly strong for those people who had greater sleep-related daytime impairment at baseline. This pattern of findings has been observed in the depression literature [69] and may reflect a floor effect for people with a lower level of symptoms at baseline.

There are several limitations. First, we did not have the resources to collect data on specific aspects of the context that are important within i-PARIHS such as the outer context nor the dynamic relationships between the micro, meso and macro layers of context [1]. Second, likely due to county-level cluster randomization and demographic differences, there were baseline differences between the Adapted and Standard TSC groups. Third, although we trained providers to deliver 8 sessions in Standard TSC, they administered an average of 3.69 sessions when considering the full sample. When focusing only on those who completed the full course of TSC, the number of TSC sessions received by patients was closer to the ideal with an average of 6.33 sessions delivered by Standard TSC providers. Perhaps delivering eight sessions in CMHCs may be unrealistic. Fourth, the study design did not allow for a comparison between TSC and UC-DT at the 6-month follow-up. Also, for Aim 3, an attempt was made to collect data from providers mid-treatment but this was largely not successful due to the large load providers carry. Hence, mid-treatment data was not used for the mediation analysis. While it has been argued that mediators and outcomes that are both measured at post-treatment may confer advantages for clinical trials, because these timepoints capture the interval during which the greatest changes are most likely to occur in the mediators and outcomes (e.g., [57–59]), we acknowledge the lack of mid-treatment data as a limitation. Related, we conducted several analyses that do not preclude different or bidirectional models. For example, for Aim 2, the reported analyses cannot rule out the possibility that providers' perceptions of treatment fit were influenced by their observation of patient improvement. However, the proposed model is consistent with implementation science frameworks that posit that fit outcomes precede patient outcomes [70] and thus is theoretically grounded. Also, for Aim 1 we acknowledge that the relationship between sleep, functional impairment, and psychiatric symptoms is likely bidirectional [71]. However, the tested intervention targets sleep, which is expected to have downstream effects on functional impairment and psychiatric symptoms. Thus, we opted to focus on these more theoretically-grounded models for the present study [72]. Fifth, as evident in Fig. 2, most Gen 2 providers trained in TSC declined to participate. Thus, we cannot rule out the possibility that the results reflect high motivation and interest from a minority of providers. Finally, it was not always possible to determine whether drop-outs were due to patient disengagement or provider limitations, underscoring the need for clearer tracking of drop-out causes in future studies.

## Conclusion

Within the infrastructure of the Implementation Phase [5, 28] of this three-part hybrid type 2 effectiveness-implementation study and with the support of facilitation, TTT was effective. Returning to the i-PARIHS framework, the results indicate that TTT can be used to train CMHC providers to deliver TSC via facilitation that is delivered by university-based external facilitators. These findings add to the growing evidence for the use of multi-component implementation strategies and external facilitation as effective approaches to promoting health-care innovations like TTT and TSC into routine practice (e.g., [37–39]). These results also contribute to the dearth of evidence collected from Generation 2 providers who had been trained by local CMHC trainers and Generation 2 patients [17, 22, 73] and add support to using a briefer version of TSC in under-resourced settings [5].

## Supplementary Information

Supplementary Material 1.

## Data Availability

Raw data for most outcomes reported herein has been uploaded into the NIMH National Data Archive.

## Abbreviations

6FU: Six months after treatment
AIM: Acceptability of Intervention Measure
ANCOVA: Analysis of covariance
CBT: Cognitive behavioral therapy
CEQ: Credibility/Expectancy Questionnaire
CMHCs: Community mental health centers
DSM-5: DSM-5 Cross-Cutting Measure
EBPT: Evidence-based psychological treatments
FIM: Feasibility of Intervention Measure
IAM: Intervention Appropriateness measure
ICC: Intraclass correlation coefficients
MDES: Minimum detectable effect sizes
MLMs: Multilevel models
MP: Mediated proportions
PROMIS-SD: PROMIS-Sleep Disturbance
PROMIS-SRI: PROMIS-Sleep Related Impairment
SDS: Sheehan Disability Scale
SE: Robust standard errors
SEMs: Structural equation models
SHC: Sleep Health Composite
SMI: Serious mental illness
SNAP: Supplemental Nutrition Assistance Program
SSI/SSDI: Supplemental Social Security Income/Social Security Disability Insurance
TSC: Transdiagnostic Intervention for Sleep and Circadian Problems
TTT: Train-the-Trainer
UC-DT: Usual Care followed by Delayed Treatment with TSC

## Footnotes

1 Previously referred to as TranS-C.

2 Individuals who are currently suicidal require a higher level of care. This could involve more frequent appointments with a case manager or psychiatrist, or admission for inpatient treatment. In such cases, study-related assessments or treatment sessions were not always feasible, and availability to participate was discussed with the patient and provider.
