# Supplementary material for: A randomized trial of adapted versus standard versions the transdiagnostic intervention for sleep and circadian dysfunction (TSC) implemented via facilitation and delivered by community mental health providers using train-the-trainer
Source: Implement Sci. 2025 Nov 29;21:5. doi: 10.1186/s13012-025-01467-y (PMC12801857; doi:10.1186/s13012-025-01467-y)
Supplement: Supplementary file 1 — Supplementary Material 1. [file 13012_2025_1467_MOESM1_ESM.docx]

**Additional File 1: Further information on data analysis**

**Power Analysis**

A pre-specified power analysis was conducted for the entire trial, which included providers and patients from the Implementation Phase (i.e., CMHC providers trained by the UC Berkeley team) (1) and the TTT Phase (i.e., CMHC providers who were trained by local trainers, who had been trained by the UC Berkeley team and the focus of the present report) (2). However, these two phases were subsequently separated to more thoroughly investigate results from each phase of the study (i.e., Implementation Phase and TTT Phase). Thus, for the present study, the minimum detectable effect sizes (MDES) were calculated with the sample from the TTT Phase for the primary patient outcome: PROMIS-SD comparing TSC and UC-DT. Optimal Design software for cluster-randomized trials with repeated measurements was used to calculate the MDES (3, 4). Intraclass correlation coefficients (ICC) were calculated for multilevel models with timepoints (level 1) nested within patients or providers (level 2) nested with CMHCs (level 3). The resulting ICCs were 0.01 for the patient outcome of sleep disturbance. For sleep disturbance, using the ICC of 0.01, the final sample size of *N* = 143 patients, alpha of 0.05, 8 CMHCs, and power = 0.80, the MDES was 0.76. Therefore, the study has sufficient power to detect an effect size of 0.76.

**Analysis Plan**

For analyses related to Aims 1 to 3 and most exploratory aims, the patient assessments for the immediate TSC condition at pre-treatment and post-treatment were used. For UC-DT, included in Aim 1 and exploratory Aim 2 only, analyses used patient assessments completed at pre-treatment and four or eight weeks after pre-treatment (i.e., at the end of usual care and before delayed treatment with TSC), depending on whether their county had been randomized to Adapted or Standard TSC, respectively. Exploratory Aim 1 from the protocol paper was not included in the current paper because it will be included as part of a longer report comparing Generation 1 and 2 on patient outcomes.

A 6-month follow-up (6FU) for immediate and delayed TSC was collected but was not included in the present analyses because the prespecified analyses for Aim 1 focus on timepoints with a comparable UC-DT comparison group. The one exception is that for the PhenX Tookit outcomes, both post-treatment and 6FU were included to examine the effects of TSC treatment condition (Adapted vs. Standard) on patient outcomes. This timepoint was only included for these analyses, because (a) it was delineated in these prespecified analyses, and (b) comparable comparisons for Adapted and Standard TSC were available at both timepoints. Analyses not comparing UC-DT to immediate TSC combined data from the delayed treatment phase of UC-DT with the immediate TSC condition. Specifically, pre-treatment data from both groups, along with post-treatment and 6FU data from the UC-DT group following TSC treatment and equivalent timepoints from the Immediate TSC group, were included in the analysis. In other words, the TSC data from both UC-DT and Immediate TSC were analyzed. The rationale for this decision is threefold 1) the treatment contents were the same for two conditions, 2) the only difference between the two conditions was that those in the UC-DT group received usual care until starting TSC after a delay period and 3) to increase sample size and achieve greater statistical power.

**Multilevel Models (Aims 1 & 2 and Exploratory Aims 1 & 2).** Analyses used intent-to-treat principles, which performs well in simulations of MLMs with missing data up to 50% (5). Models comparing UC-DT versus TSC used maximum likelihood estimation (MLE) to handle missing data under the missing at random assumption and robust standard errors. Models comparing Adapted and Standard TSC used restricted maximum likelihood estimation (REML) to handle missing data with the Kenward-Roger approximation given the small patient sample size in the Standard TSC condition (6). In simulation MLM studies, these approaches have been found to adequately minimize bias with as few as five clusters (7). In the present study, we had at least 7 clusters per condition. Effect sizes for all multilevel models are represented with ‘*d*’ and were calculated following Feingold (8, equation 5), using unadjusted change scores and raw standard deviations at pre-treatment from each treatment condition. The Benjamini-Hochberg procedure (9) was used for the primary outcome (sleep disturbance) to control the false discovery rate, per the protocol paper. Note that, in the protocol paper, the Sleep Health Composite was listed in the Measures section but was omitted from the planned analysis section in error. For Exploratory Aim 1, average cigarettes per day among people who endorsed using tobacco was included as an outcome for the Implementation Phase (10), but there was not enough variability in this outcome in the TTT Phase. Therefore, it was not included as an outcome.

**Linear Regression Models (Aim 3 and Exploratory Aim 1).** For Aim 3**,** models adjusted for the relevant fit at post-training and outcome variable at pre-treatment. Effect sizes for linear regressions are partial eta squared, or the proportion of variance explained by the predictor of interest (11). For all linear regression models, Partial eta^2^ is reported as an effect size and can be interpreted as the proportion of variance explained by each predictor (11).

**Structural Equation Modeling (SEM) (Aim 1).** The analysis of covariance (ANCOVA) approach was used, in which pre-treatment measures of the mediator and outcome are included as covariates.^[[1]](#footnote-1)^ This approach has been recommended for designs comparing pre- to post-treatment (12). In particular, statisticians and methodologists have argued that contemporaneous models, whereby the mediators and outcomes are both measured at post-treatment, may confer advantages for clinical trials, because these timepoints capture the interval during which the greatest changes are most likely to occur in the mediators and outcomes (e.g., 12, 13, 14). Also, TSC only targets sleep and circadian processes so any change in mental health or functioning is likely due to improvement in sleep and circadian functioning. The predictor was condition (immediate TSC vs. UC-DT), the mediator was PROMIS-SD or PROMIS-SRI at post-treatment, and the outcomes were DSM-5 Cross-Cutting and SDS at post-treatment. For all SEMs, the parameter of interest was the indirect effect. Maximum likelihood estimation was used. As noted above, all models were run with robust standard errors. Effect sizes for mediation models are the mediated proportions (MP), or the proportion of the total effect that is explained by the indirect effect expressed as a percentage (15).

**Assumptions Checks**

Model assumptions for multilevel models (MLMs), structural equation models (SEMs), and linear regressions models were evaluated. For MLMs and linear regressions, qqplots and histograms of model residuals were examined. Additionally, Levene’s test was used to formally test for homogeneity. For SEMs, most models were saturated, resulting in a null residual matrix. Thus, the normality and homogeneity of the residuals for each path were evaluated. In some models, violations of normality and homoscedasticity were detected. Therefore, across most models, robust standard errors (‘sandwich estimators’) were used, which are robust to violations of assumptions, particularly homoscedasticity (Rabe-Hesketh & Skrondal, 2011). The one exception was that for MLM models comparing the effects of TSC condition (Adapted versus Standard), restricted maximum likelihood (REML) and Kenward-Roger approximation were prioritized to adjust for the small sample size in the Standard TSC condition. The latter models were also run using robust standard errors in case these different approaches changed the pattern of results. Most models did not differ when using robust standard errors or REML and the Kenward-Roger method, but any differences that were observed have been noted in the relevant results table (Additional File Tables 8 and 10 below).

**Missing Data**

Per the protocol paper, we tested whether missingness was related to other, theoretically plausible predictors, which would suggest that the data were missing at random (Black et al., 2017; Grund et al., 2019; Rabe-Hesketh & Skrondal, 2012). First, we evaluated whether the following observed variables predicted missingness of *patient* data at post-treatment: treatment condition (UC-DT vs. immediate and Adapted vs. Standard; Rabe-Hesketh & Skrondal, 2012), patient sex (given its relationship to sleep problems; e.g., Zhang & Wing, 2006), other patient sociodemographics (e.g., income, education, race, ethnicity, number of children, government assistance, employment status; Hale et al., 2020), patient variables used for stratification (age, substance use, psychosis; Kahan & Morris, 2012), and provider theoretical orientation (CBT or other; e.g., Addis & Krasnow, 2000; Garcia et al., 2020), prior training in sleep problems (Garcia et al., 2020), and perceptions of TSC fit at pre, mid, and post (given that treatment fit can predict providers’ ongoing use of a given treatment; e.g., Barnett et al., 2021). Second, we tested whether the following observed variables predicted missingness of *provider* data at post-treatment: treatment condition (Adapted vs. Standard; Rabe-Hesketh & Skrondal, 2012), variables that have been found to predict provider perceptions or use of treatment (i.e., CBT orientation, prior training in sleep problems; Addis & Krasnow, 2000; Garcia et al., 2020), and patient symptom severity at pre-treatment (based on qualitative data that this impacted provider use or discontinuation of TSC; Sarfan et al., 2024). Third, we tested whether the planned covariate of county predicted missingness for patients and providers (Callaway et al., 2023). To test these possibilities, we created a dummy-coded variable for all patients to indicate whether they had missing data or no missing data at post-treatment (1 = missing data; 0 = no missing data). We then used logistic regression or chi-square tests, depending on whether the predictor was continuous or categorical, respectively, to test whether any of these observed variables predicted missingness group membership (i.e., whether patients had missing data or did not have missing data).

Findings from these analyses suggested that patients’ treatment condition assignments were associated with missing data at post-treatment, such that patients in UC-DT (vs. immediate TSC) had less missing data. Patients who were not Hispanic or Latino (vs. Hispanic or Latino) had less missing data. For providers, treatment condition predicted missing data, such that Adapted (vs. Standard) was associated with less missing data. Providers who had a marriage and family therapy degree (vs. other degrees) had more missing data. These results for providers and patients lend support to the data being missing at random. Specifically, although the missing at random assumption is impossible to statistically verify (16), our missing data appeared to be associated with theoretically-grounded observed variables (Black et al., 2017; Grund et al., 2019; Rabe-Hesketh & Skrondal, 2012). County was found to predict missing data for both patients and providers. As specified in the protocol paper, these variables were included in the relevant models as covariates, except when they were already tested as predictors in a given model.

***Covariates***

Per the protocol paper, covariates tested included planned variables and variables identified during the aforementioned missing data analyses (Callaway et al., 2023). For Aims 1 and 2 and Exploratory Aims 1 and 2, the covariates included patient TSC condition (UC-DT vs. Immediate; *identified from missing data analyses)*, patient stratification variables (age, substance use, psychosis; *planned*), patient ethnicity *(from missing data analyses)*, and county *(planned)*. For Aim 3, the covariates were the same as Aim 1 but also included the provider covariates that predicted provider missing data, TSC condition (Standard vs. Adapted; *from missing data analyses*) and provider degree *(from missing data analyses)*.

For Aim 2 and exploratory Aim 1, there was collinearity between one level of county and TSC condition (Standard vs. Adapted). As TSC condition was a predictor in these models, county was removed as a covariate. For these aims there were no differences in models whether or not county was included. For Aim 3, there was collinearity between county and both provider covariates (TSC condition and provider degree). As county was a planned covariate and predicted missing data, county was retained as a covariate and the two provider covariates were removed from the models. Differences when including county versus the provider covariates are noted in Table 7.

**Patient and Provider descriptives**

Relative to completers, participants who did not begin treatment or who dropped out were not significantly different on the stratification factors of sex (*χ^2^* < 0.01, df = 1, *p* = 1.00), age group (above or below 50 years; *χ^2^* = 1.09, df = 1, *p* = 0.30) or psychosis status (*χ^2^* = 0.39, df = 1, *p* = 0.53).

As evident in Table 1, Adapted and Standard TSC did not differ on any pre-treatment patient demographic variable except on race (*p* = 0.05), civil status (*p* = 0.04), and annual household income (*p* = 0.05). Specifically, for race, more patients in Standard endorsed other/category not listed (25.00%) than in Adapted (4.72%). For civil status, more patients in Standard endorsed other/category not listed (6.3%) than in Adapted (0.00%), while for income, a greater number in Standard reported an annual household income of $20,000 - $30,000 (31.3%) than in Adapted (5.51%). There was no difference between Adapted and Standard TSC on the number of treatment sessions completed (*p* = 0.48). As evident in Table 2, Adapted and Standard TSC did not differ on any provider demographic variable except on race (*p* = 0.05) and employment duration (*p =* 0.001). More providers in Standard were Black or African American (33.3%) than in Adapted (2.1%). Employment duration of providers was significantly longer in Adapted (*M* = 5.41 years) than in Standard (*M* = 1.20 years). Additional File 1, Supplement Table 9 presents the patient demographics by immediate TSC vs. UC-DT condition, which did not differ on any pre-treatment patient demographic variable except that ethnicity (*p* = 0.07) approached significance.

**Pre-specified Analyses from Protocol Paper**

Aims 2 and 3 that were pre-specified in the protocol paper (Callaway et al., 2023) were not reported in the main text due to the small sample size in the Standard condition for the provider perceived fit variables at post-treatment (*N* = 1). To increase the sample size TSC data from the delayed treatment phase of UC-DT were combined with data from the immediate TSC condition resulting in *N* = 4. For comprehensiveness the results are reported here. However, the results should be interpreted with much caution as the very small sample size likely resulted in unreliable estimates.

Aim 2, as specified in the protocol paper, was to evaluate whether TSC treatment condition (Adapted vs. Standard TSC) was associated with fit to the CMHC context, operationalized as provider ratings of acceptability, appropriateness, and feasibility, for Generation 2 providers. We hypothesized that Adapted TSC would be superior to Standard TSC with respect to the primary provider outcome of acceptability and the secondary provider outcomes of appropriateness and feasibility. Aim 3 specified in the protocol paper was to evaluate whether perceived fit among Generation 2 providers mediated the relation between TSC treatment condition and Generation 2 patient outcomes. We hypothesized that relative to Standard TSC, Adapted TSC would be associated with greater reductions in the primary and secondary patient outcomes indirectly through higher provider ratings of acceptability, appropriateness, and feasibility. Exploratory analyses focused on: (1) comparing whether the effectiveness of TSC for primary and secondary patient outcomes is moderated by generation, (2) comparing Adapted and Standard TSC on Generation 2 patient perceptions of credibility and perceived improvement and select PhenX Toolkit outcomes that are both strongly related to SMI and sleep and circadian problems (e.g., substance use, suicidality) (17, 18) and of high priority to the community partners who participated; and (3) determining whether treatment effects for Generation 2 patients are moderated by risk factors including age, sex, and sleep and circadian and psychiatric symptoms at baseline. In particular, emerging evidence suggests that patients who are older and have more severe sleep and circadian and psychiatric symptoms at baseline demonstrate poorer response to sleep and circadian treatment, whereas outcomes by sex have been mixed for patients with SMI (e.g., 19, 20).

***Power Analysis (Protocol Paper)***

The intraclass correlation coefficient (ICC) was calculated for multilevel models with timepoints (level 1) nested within patients or providers (level 2) nested with CMHCs (level 3). The resulting ICC was < 0.0001 for the provider outcome of acceptability. To be conservative an ICC of 0.001 was used for providers. For acceptability, using an ICC of 0.001, the final sample size of *N* = 53 providers, alpha of 0.05, 8 CMHCs, and power = 0.80, the MDES was 1.00.

***Analyses***

**Multilevel Models (Protocol Paper, Aim 2)** For Aim 2 specified in the protocol paper, the same MLM approach as Aims 1 and 2 and Exploratory Aims 1 and 2 reported in the main text herein was used. For Aim 2 specified in the protocol paper, the predictor was TSC condition (Standard vs. Adapted). The outcomes were providers’ perceptions of acceptability, feasibility, and appropriateness.

**SEM Models (Protocol Paper, Aim 3)** For Aim 3 specified in the protocol paper, the approach mirrored that of SEM models conducted for Aim 1 reported in the main text herein. The predictor was TSC condition (Adapted vs. Standard), the mediator was AIM, FIM, or IAM at post-treatment, and the outcomes were PROMIS-SD, PROMIS-SRI, DSM-5 Cross-Cutting, and SDS at post-treatment and six-month follow-up.

***Covariates (Protocol Paper Aims 2 and 3)***

Original Protocol Aims 2 and 3 included patient and provider covariates that were determined from the missing data analyses. In both Aims, co-linearity prevented county running with the predictor TSC condition (Standard vs. Adapted) in each model. County was removed as a covariate for these models. For Original Protocol Aim 2 there were no differences in results whether including county or not. For Protocol Aim 3, models would not run when county was included as a covariate and, thus, it was not included.

**Protocol Paper Aim 2 Results**

See Supplement Table 1 below for Original Protocol Aim 2 MLM results. TSC condition (Standard versus Adapted) was not significantly associated with the change in providers’ perceptions of acceptability, feasibility, and appropriateness from pre- to post-treatment.

**Protocol Paper Aim 3 Results**

See Supplement Table 2 below for path coefficients and standard errors of Original Protocol Aim 3 SEMs. There were no significant indirect effects of acceptability, appropriateness, and feasibility on the relations between TSC condition (Adapted versus Standard) and the primary or secondary patient outcomes of sleep disturbance, sleep-related impairment, psychiatric symptoms, or overall functional. The indirect effects explained 2.03% to 419.72% of the total effects for each of these models. However, the large mediated proportions in some of these models are likely the result of the very small sample used in these models.

**Supplement Table 1. Original Protocol Aim 2: Multilevel Modeling Results for TSC Condition (Standard vs. Adapted) on Provider Perceptions of Treatment Fit from Pre- to Post-Treatment**

|  | ***b*** | **SE** | ***p* value** |
| --- | --- | --- | --- |
| **Outcome** |  |  |  |
| AIM | -0.17 | 0.29 | 0.57 |
| FIM | 0.39 | 0.39 | 0.31 |
| IAM | -0.22 | 0.35 | 0.54 |
| *Note. b* = time-by-treatment interaction. SE = standard errors. AIM = Acceptability of Intervention Measure. FIM = Feasibility of Intervention Measure. IAM = Intervention Appropriateness measure. When using robust SEs (vs. REML & Kenward-Roger) there was a significant effect of TSC Condition (Adapted vs. Standard) on FIM from Pre- to Post-Treatment (*b* = 0.36, *SE* = 0.17, *p* = 0.03) such that provider ratings of feasibility significantly decreased from pre- to post-treatment only in the Standard TSC condition. | | | |

**Supplement Table 2. Original Protocol Aim 3: Mediation Models of Acceptability, Feasibility, and Appropriateness on Relations between TSC Condition (Standard vs. Adapted) and Outcome at POST and 6FU**

|  | coefficient | SE | z | *p* | 95% Confidence Interval of effect | %MP |
| --- | --- | --- | --- | --- | --- | --- |
| **Aim 3 Model 1: TSC 🡪 Acceptability 🡪 PROMIS-SD POST** | | | | | |  |
| Path a | -0.35 | 0.19 | -1.91 | 0.06 | -0.72, 0.01 | - |
| Path b | -5.74 | 3.71 | -1.54 | 0.12 | -13.02, 1.54 | - |
| Total effect | 3.86 | 9.47 | 0.41 | 0.68 | -14.70, 22.41 | - |
| Indirect effect | 2.04 | 1.73 | 1.17 | 0.24 | -1.36, 5.43 | 52.85% |
| **Aim 3 Model 2: TSC 🡪 Acceptability 🡪 PROMIS-SD 6FU** | | | | | |  |
| Path a | -0.35 | 0.19 | -1.91 | 0.06 | -0.72, 0.01 | - |
| Path b | -3.12 | 2.51 | -1.25 | 0.21 | -8.03, 1.79 | - |
| Total effect | 3.01 | 4.15 | 0.73 | 0.47 | -5.11, 11.14 | - |
| Indirect effect | 1.11 | 1.04 | 1.06 | 0.29 | -0.93, 3.15 | 36.88% |
| **Aim 3 Model 3: TSC 🡪 Acceptability 🡪 PROMIS-SRI POST** | | | | | |  |
| Path a | -0.37 | 0.17 | -2.13 | 0.03 | -0.71, -0.03 | - |
| Path b | -1.49 | 3.97 | -0.38 | 0.71 | -9.28, 6.29 | - |
| Total effect | -2.83 | 8.47 | -0.33 | 0.74 | -19.43, 13.76 |  |
| Indirect effect | 0.55 | 1.52 | 0.36 | 0.72 | -2.43, 3.53 | 19.43% |
| **Aim 3 Model 4: TSC 🡪 Acceptability 🡪 PROMIS-SRI 6FU** | | | | | |  |
| Path a | -0.37 | 0.17 | -2.13 | 0.03 | -0.71, -0.03 | - |
| Path b | -7.06 | 2.94 | -2.40 | 0.02 | -12.83, -1.30 | - |
| Total effect | 5.47 | 5.53 | 0.99 | 0.32 | -5.36, 16.30 | - |
| Indirect effect | 2.60 | 1.64 | 1.59 | 0.11 | -0.61, 5.82 | 47.53% |
| **Aim 3 Model 5: TSC 🡪 Acceptability 🡪 DSM-5 Cross-Cutting POST** | | | | | |  |
| Path a | -0.39 | 0.19 | -2.04 | 0.04 | -0.76, -0.01 | - |
| Path b | -2.33 | 3.11 | -0.75 | 0.45 | -8.42, 3.76 | - |
| Total effect | 2.77 | 4.42 | 0.63 | 0.53 | -5.90, 11.43 | - |
| Indirect effect | 0.90 | 1.22 | 0.74 | 0.46 | -1.49, 3.29 | 32.49% |
| **Aim 3 Model 6: TSC 🡪 Acceptability 🡪 DSM-5 Cross-Cutting 6FU** | | | | | |  |
| Path a | -0.39 | 0.19 | -2.04 | 0.04 | -0.76, -0.01 | - |
| Path b | -4.97 | 2.14 | -2.32 | 0.02 | -9.16, -0.77 | - |
| Total effect | 4.93 | 4.57 | 1.08 | 0.28 | -4.03, 13.88 | - |
| Indirect effect | 1.91 | 1.15 | 1.67 | 0.10 | -0.34, 4.17 | 38.74% |
| **Aim 3 Model 7: TSC 🡪 Acceptability 🡪 SDS POST** | | | | | | |
| Path a | -0.38 | 0.18 | -2.12 | 0.03 | -0.72, -0.03 | - |
| Path b | -0.48 | 1.87 | -0.26 | 0.80 | -4.15, 3.19 | - |
| Total effect | 1.34 | 2.46 | 0.55 | 0.59 | -3.48, 6.16 | - |
| Indirect effect | 0.18 | 0.72 | 0.25 | 0.80 | -1.22, 1.59 | 13.43% |
| **Aim 3 Model 8: TSC 🡪 Acceptability 🡪 SDS 6FU** | | | | | | |
| Path a | -0.38 | 0.18 | -2.12 | 0.03 | -0.72, -0.03 | - |
| Path b | -1.59 | 1.95 | -0.81 | 0.42 | -5.42, 2.25 | - |
| Total effect | 4.53 | 2.50 | 1.81 | 0.07 | -0.37, 9.43 | - |
| Indirect effect | 0.60 | 0.79 | 0.76 | 0.45 | -0.95, 2.14 | 13.25% |
| **Aim 3 Model 9: TSC 🡪 Appropriateness 🡪 PROMIS-SD POST** | | | | | | |
| Path a | -0.25 | 0.32 | -0.76 | 0.45 | -0.88, 0.39 | - |
| Path b | -4.93 | 2.45 | -2.01 | 0.04 | -9.73, -0.13 | - |
| Total effect | 1.56 | 9.68 | 0.16 | 0.87 | -17.41, 20.54 | - |
| Indirect effect | 1.22 | 1.69 | 0.72 | 0.47 | -2.09, 4.53 | 78.21% |
| **Aim 3 Model 10: TSC 🡪 Appropriateness 🡪 PROMIS-SD 6FU** | | | | | | |
| Path a | -0.25 | 0.32 | -0.76 | 0.45 | -0.88, 0.39 | - |
| Path b | -0.94 | 1.96 | -0.48 | 0.63 | -4.78, 2.90 | - |
| Total effect | 2.36 | 4.18 | 0.56 | 0.57 | -5.83, 10.54 | - |
| Indirect effect | 0.23 | 0.57 | 0.41 | 0.68 | -0.88, 1.35 | 9.75% |
| **Aim 3 Model 11: TSC 🡪 Appropriateness 🡪 PROMIS-SRI POST** | | | | | | |
| Path a | -0.31 | 0.34 | -0.93 | 0.36 | -0.97, 0.35 | - |
| Path b | 1.79 | 2.66 | 0.67 | 0.50 | -3.42, 7.00 | - |
| Total effect | -2.95 | 8.76 | -0.34 | 0.74 | -20.11, 14.22 | - |
| Indirect effect | -0.55 | 1.05 | -0.53 | 0.60 | -2.61, 1.50 | 18.64% |
| **Aim 3 Model 12: TSC 🡪 Appropriateness 🡪 PROMIS-SRI 6FU** | | | | | | |
| Path a | -0.31 | 0.34 | -0.93 | 0.36 | -0.97, 0.35 | - |
| Path b | -1.88 | 2.15 | -0.87 | 0.38 | -6.08, 2.33 | - |
| Total effect | 5.22 | 5.64 | 0.93 | 0.36 | -5.84, 16.28 | - |
| Indirect effect | 0.58 | 0.89 | 0.65 | 0.52 | -1.17, 2.33 | 11.11% |
| **Aim 3 Model 13: TSC 🡪 Appropriateness 🡪 DSM-5 POST** | | | | | | |
| Path a | -0.32 | 0.34 | -0.96 | 0.34 | -0.98, 0.34 | - |
| Path b | -4.30 | 1.83 | -2.35 | 0.02 | -7.89, -0.71 | - |
| Total effect | 0.68 | 4.74 | 0.14 | 0.89 | -8.61, 9.98 | - |
| Indirect effect | 1.39 | 1.48 | 0.94 | 0.35 | -1.52, 4.29 | 204.41% |
| **Aim 3 Model 14: TSC 🡪 Appropriateness 🡪 DSM-5 6FU** | | | | | | |
| Path a | -0.32 | 0.34 | -0.96 | 0.34 | -0.98, 0.34 | - |
| Path b | -4.53 | 1.31 | -3.46 | 0.001 | -7.10, -1.97 | - |
| Total effect | 4.07 | 4.79 | 0.85 | 0.40 | -5.31, 13.45 | - |
| Indirect effect | 1.46 | 1.55 | 0.94 | 0.35 | -1.58, 4.51 | 35.87% |
| **Aim 3 Model 15: TSC 🡪 Appropriateness 🡪 SDS POST** | | | | | | |
| Path a | -0.34 | 0.34 | -1.00 | 0.32 | -1.00, 0.33 | - |
| Path b | -0.95 | 1.32 | -0.72 | 0.47 | -3.53, 1.64 | - |
| Total effect | 0.11 | 2.59 | 0.04 | 0.97 | -4.97, 5.18 | - |
| Indirect effect | 0.32 | 0.57 | 0.57 | 0.57 | -0.79, 1.43 | 290.91% |
| **Aim 3 Model 16: TSC 🡪 Appropriateness 🡪 SDS 6FU** | | | | | | |
| Path a | -0.34 | 0.34 | -1.00 | 0.32 | -1.00, 0.33 | - |
| Path b | 0.42 | 1.44 | 0.29 | 0.77 | -2.40, 3.24 | - |
| Total effect | 4.43 | 2.45 | 1.81 | 0.07 | -0.36, 9.22 | - |
| Indirect effect | -0.14 | 0.52 | -0.28 | 0.78 | -1.16, 0.87 | 3.16% |
| **Aim 3 Model 17: TSC 🡪 Feasibility 🡪 PROMIS-SD POST** | | | | | | |
| Path a | 0.36 | 0.37 | 0.96 | 0.34 | -0.37, 1.09 | - |
| Path b | -8.33 | 2.14 | -3.88 | <0.001 | -12.53, -4.12 | - |
| Total effect | -0.71 | 9.73 | -0.07 | 0.94 | -19.79, 18.36 | - |
| Indirect effect | -2.98 | 3.27 | -0.91 | 0.36 | -9.38, 3.42 | 419.72% |
| **Aim 3 Model 18: TSC 🡪 Feasibility 🡪 PROMIS-SD 6FU** | | | | | | |
| Path a | 0.36 | 0.37 | 0.96 | 0.34 | -0.37, 1.09 | - |
| Path b | -3.14 | 1.97 | -1.59 | 0.11 | -7.00, 0.73 | - |
| Total effect | 1.38 | 4.30 | 0.32 | 0.75 | -7.04, 9.80 | - |
| Indirect effect | -1.12 | 1.36 | -0.83 | 0.41 | -3.78, 1.54 | 81.16% |
| **Aim 3 Model 19: TSC 🡪 Feasibility 🡪 PROMIS-SRI POST** | | | | | | |
| Path a | 0.28 | 0.36 | 0.76 | 0.45 | -0.44, 0.99 | - |
| Path b | -3.37 | 2.46 | -1.37 | 0.17 | -8.20, 1.46 | - |
| Total effect | -5.27 | 8.76 | -0.60 | 0.55 | -22.44, 11.90 | - |
| Indirect effect | -0.94 | 1.41 | -0.66 | 0.51 | -3.71, 1.83 | 17.84% |
| **Aim 3 Model 20: TSC 🡪 Feasibility 🡪 PROMIS-SRI 6FU** | | | | | | |
| Path a | 0.28 | 0.36 | 0.76 | 0.45 | -0.44, 0.99 | - |
| Path b | -2.04 | 2.22 | -0.92 | 0.36 | -6.39, 2.32 | - |
| Total effect | 4.64 | 5.64 | 0.82 | 0.41 | -6.41, 15.69 | - |
| Indirect effect | -0.57 | 0.96 | -0.59 | 0.55 | -2.44, 1.31 | 12.28% |
| **Aim 3 Model 21: TSC 🡪 Feasibility 🡪 DSM-5 Cross-Cutting POST** | | | | | | |
| Path a | 0.32 | 0.38 | 0.84 | 0.40 | -0.42, 1.07 | - |
| Path b | -4.12 | 1.98 | -2.08 | 0.04 | -8.00, -0.24 | - |
| Total effect | 0.36 | 4.58 | 0.08 | 0.94 | -8.62, 9.34 | - |
| Indirect effect | -1.32 | 1.70 | -0.78 | 0.44 | -4.65, 2.01 | 366.67% |
| **Aim 3 Model 22: TSC 🡪 Feasibility 🡪 DSM-5 Cross-Cutting 6FU** | | | | | | |
| Path a | 0.32 | 0.38 | 0.84 | 0.40 | -0.42, 1.07 | - |
| Path b | -4.63 | 1.68 | -2.76 | 0.01 | -7.92, -1.34 | - |
| Total effect | 4.17 | 4.74 | 0.88 | 0.38 | -5.13, 13.46 | - |
| Indirect effect | -1.49 | 1.85 | -0.80 | 0.42 | -5.11, 2.14 | 35.73% |
| **Aim 3 Model 23: TSC 🡪 Feasibility 🡪 SDS POST** | | | | | | |
| Path a | 0.27 | 0.36 | 0.74 | 0.46 | -0.44, 0.98 | - |
| Path b | -1.33 | 1.20 | -1.11 | 0.27 | -3.67, 1.02 | - |
| Total effect | -0.36 | 2.40 | -0.15 | 0.88 | -5.06, 4.34 | - |
| Indirect effect | -0.35 | 0.59 | -0.60 | 0.55 | -1.50, 0.79 | 97.22% |
| **Aim 3 Model 24: TSC 🡪 Feasibility 🡪 SDS 6FU** | | | | | | |
| Path a | 0.27 | 0.36 | 0.74 | 0.46 | -0.44, 0.98 | - |
| Path b | -0.35 | 1.50 | -0.23 | 0.82 | -3.30, 2.60 | - |
| Total effect | 4.43 | 2.48 | 1.79 | 0.07 | -0.43, 9.30 | - |
| Indirect effect | -0.09 | 0.42 | -0.23 | 0.82 | -0.91, 0.72 | 2.03% |
| *Note.* "-" indicates that value is not relevant to model. %MP = mediated proportion (i.e., the proportion of the total effect that is explained by the indirect effect expressed as a percentage). TSC = TSC treatment condition (Standard versus Adapted). PROMIS-SD = PROMIS Sleep Disturbance. PROMIS-SRI = PROMIS Sleep-Related Impairment. DSM-5 = DSM-5 Cross-Cutting. SDS = Sheehan Disability Scale. POST = post-treatment assessment. 6FU = six-month follow-up assessment. Path a = path from the independent variable to mediator (i.e., TSC condition 🡪 acceptability, appropriateness, or feasibility). Path b = path from the mediator to the outcome (acceptability, appropriateness, or feasibility 🡪 PROMIS-SD, PROMIS-SRI, DSM-5 Cross-Cutting, or SDS). All models adjust for pre-treatment levels of the relevant mediator (i.e., acceptability, appropriateness, or feasibility) and relevant outcome (i.e., PROMIS-SD, PROMIS-SRI, DSM-5 Cross-Cutting, or SDS).  **Supplement Table 3. Differences in pre-treatment intent-to-treat sample sizes for the pre-specified analyses planned in the protocol paper (Callaway et al., 2023) and sample sizes used in the final analyses.**   \|  \| Pre-specified in Protocol \| \| Final analyses \| \| \| --- \| --- \| --- \| --- \| --- \| \| **Aim** \|  \|  \|  \|  \| \| Aim 1 \| UC-DT (*n =* 65) \| Immediate (*n =* 78) \| UC-DT (*n =* 65) \| Immediate (*n =* 78) \| \| Aim 2 \| Standard (*n* = 8) \| Adapted (*n* = 70) \| Standard (*n* = 16) \| Adapted (*n* = 127) \| \| Aim 3 \| Standard (*n* = 8) \| Adapted (*n* = 70) \| Total (*n* = 143)* \| \| \| Exploratory Aim 1 \| Standard (*n* = 8) \| Adapted (*n* = 70) \| Standard (*n* = 16) \| Adapted (*n* = 127) \| \| Exploratory Aim 2 \| UC-DT (*n =* 65) \| Immediate (*n =* 78) \| UC-DT (*n =* 65) \| Immediate (*n =* 78) \| \| *The final analyses for Aim 3 uses provider fit as a continuous predictor i.e., did not compare Standard vs. Adapted TSC. \| \| \| \| \| | | | | | | |

**Supplement Table 4. Percent of Patient Missing Data for Aim 1 Outcome Measures by Treatment Condition (UC-DT vs. TSC) and Timepoint**

|  | UC-DT (*n* = 65) | | | | | | TSC (*n* = 78) | | | | Total (*N* = 143) | | | | |  |  |
| --- | --- | --- | --- | --- | --- | --- | --- | --- | --- | --- | --- | --- | --- | --- | --- | --- | --- |
|  | Pre | | Post | | | Pre | | | Post | | Pre | | Post | | | |  |
|  | n | % | n | % | n | | | % | n | % | n | % | | n | % | | |
| **Outcome** |  |  |  |  |  | | |  |  |  |  |  | |  |  | | |
| PROMIS-SD | 3 | 4.62 | 8 | 12.31 | 6 | | | 7.69 | 29 | 37.18 | 9 | 6.29 | | 37 | 25.87 | | |
| PROMIS-SRI | 0 | 0 | 8 | 12.31 | 0 | | | 0 | 29 | 37.18 | 0 | 0 | | 37 | 25.87 | | |
| SHC | 11 | 16.92 | 14 | 21.54 | 11 | | | 14.1 | 32 | 41.03 | 22 | 15.38 | | 46 | 32.17 | | |
| SDS | 0 | 0 | 8 | 12.31 | 0 | | | 0 | 29 | 37.18 | 0 | 0 | | 37 | 25.87 | | |
| DSM-5 | 0 | 0 | 8 | 12.31 | 1 | | | 1.28 | 29 | 37.18 | 1 | 0.7 | | 37 | 25.87 | | |
| *Note.* PROMIS-SD = PROMIS Sleep Disruption. PROMIS-SD = PROMIS Sleep Disturbance. PROMIS-SRI = PROMIS Sleep-Related Impairment. SHC = Sleep Health Composite. DSM-5 = DSM-5 Cross-Cutting. SDS = Sheehan Disability Scale. TSC = Transdiagnostic Intervention for Sleep and Circadian Dysfunction. UC-DT = usual care followed by delayed treatment with TSC. | | | | | | | | | | | | | | | |  |  |

**Supplement Table 5. Percent of Patient Missing Data for Aims 2 and 3 Outcome Measures by TSC Treatment Condition (Standard vs. Adapted) and Timepoint**

|  | Adapted (*n* = 127) | | | | | | Standard (*n* = 16) | | | | Total (*N* = 143) | | | | |  |  |
| --- | --- | --- | --- | --- | --- | --- | --- | --- | --- | --- | --- | --- | --- | --- | --- | --- | --- |
|  | Pre | | Post | | | Pre | | | Post | | Pre | | Post | | | |  |
|  | n | % | n | % | n | | | % | n | % | n | % | | n | % | | |
| **Outcome** |  |  |  |  |  | | |  |  |  |  |  | |  |  | | |
| PROMIS-SD | 8 | 6.3 | 51 | 40.16 | 1 | | | 6.25 | 9 | 56.25 | 9 | 6.29% | | 60 | 41.96 | | |
| PROMIS-SRI | 0 | 0 | 51 | 40.16 | 0 | | | 0 | 9 | 56.25 | 0 | 0.00% | | 60 | 41.96 | | |
| SHC | 19 | 14.96 | 55 | 43.31 | 3 | | | 18.75 | 10 | 62.5 | 22 | 15.38% | | 65 | 45.45 | | |
| SDS | 0 | 0 | 51 | 40.16 | 0 | | | 0 | 9 | 56.25 | 0 | 0.00% | | 60 | 41.96 | | |
| DSM-5 | 1 | 0.79 | 52 | 40.94 | 0 | | | 0 | 9 | 56.25 | 1 | 0.70% | | 61 | 42.66 | | |
| *Note.* PROMIS-SD = PROMIS Sleep Disruption. PROMIS-SD = PROMIS Sleep Disturbance. PROMIS-SRI = PROMIS Sleep-Related Impairment. SHC = Sleep Health Composite. DSM-5 = DSM-5 Cross-Cutting. SDS = Sheehan Disability Scale. TSC = Transdiagnostic Intervention for Sleep and Circadian Dysfunction. UC-DT = usual care followed by delayed treatment with TSC. | | | | | | | | | | | | | | | |  |  |

**Supplement Table 6. Percent of Provider Missing Data for Aim 3 and Original Protocol Aim 2 Measures by Treatment Condition (Standard vs. Adapted) and Timepoint**

|  | Adapted | | | | Standard | | | | Total | | | | |
| --- | --- | --- | --- | --- | --- | --- | --- | --- | --- | --- | --- | --- | --- |
|  | Pre  (*n* = 47) | | Post  (*n* = 127) | | Pre  (*n* = 6) | | Post  (*n* = 16) | | Pre  (*n* = 53) | | Post  (*n* = 143) | |  |
|  | n | % | n | % | n | % | n | % | n | % | n | % |  |
| **Outcome** |  |  |  |  |  |  |  |  |  |  |  |  |  |
| AIM | 12 | 25.53 | 57 | 44.88 | 1 | 16.67 | 12 | 75 | 13 | 24.53 | 69 | 48.25 |  |
| IAM | 12 | 25.53 | 57 | 44.88 | 1 | 16.67 | 12 | 75 | 13 | 24.53 | 69 | 48.25 |  |
| FIM | 12 | 25.53 | 57 | 44.88 | 1 | 16.67 | 12 | 75 | 13 | 24.53 | 69 | 48.25 |  |
| *Note.* AIM = Acceptability of Intervention Measure. FIM = Feasibility of Intervention Measure. IAM = Intervention Appropriateness measure. ‘Standard’ and ‘Adapted’ indicate TSC condition. Each provider only completed the pre-treatment AIM, IAM, FIM one time. They were asked to complete these measures at post for each patient (which is why the post-treatment sample sizes are larger than pre-treatment). Providers were not asked to complete these measures at 6FU. | | | | | | | | | | | | | |

**Supplement Table 7. Percent of Missing Data for Original Protocol Aim 3 Measures by TSC Treatment Condition (Standard vs. Adapted) and Timepoint**

|  | Adapted | | | | | | Standard | | | | | | | | Total | | | | | | | | |
| --- | --- | --- | --- | --- | --- | --- | --- | --- | --- | --- | --- | --- | --- | --- | --- | --- | --- | --- | --- | --- | --- | --- | --- |
| **Provider Outcomes** | Pre  (n = 47) | | Post  (n = 127) | | 6FU  (n = 127) | | Pre  (n = 6) | | Post  (n = 16) | | | 6FU (n = 16) | | | Pre  (n = 53) | | | Post  (n = 143) | | | 6FU  (n = 143) | | |
|  | *n* | % | *n* | % | *n* | % | *n* | % | *n* | % | *n* | | % | *n* | | % | *n* | | % | *n* | | % | |
| AIM | 12 | 25.53 | 57 | 44.88 | n/a | n/a | 1 | 16.67 | 12 | 75 | n/a | | n/a | 13 | | 24.53 | 69 | | 48.25 | n/a | | n/a | |
| IAM | 12 | 25.53 | 57 | 44.88 | n/a | n/a | 1 | 16.67 | 12 | 75 | n/a | | n/a | 13 | | 24.53 | 69 | | 48.25 | n/a | | n/a | |
| FIM | 12 | 25.53 | 57 | 44.88 | n/a | n/a | 1 | 16.67 | 12 | 75 | n/a | | n/a | 13 | | 24.53 | 69 | | 48.25 | n/a | | n/a | |
| **Patient Outcomes** | Pre  (n = 127) | | Post  (n = 127) | | 6FU  (n = 127) | | Pre  (n = 16) | | Post  (n = 16) | | | 6FU  (n = 16) | | | Pre  (n = 143) | | | Post  (n = 143) | | | 6FU  (n = 143) | | |
|  | *n* | % | *n* | % | n | % | *n* | % | *n* | % | *n* | | % | *n* | | % | *n* | | % | *n* | | % | |
| PROMIS-SD | 8 | 6.3 | 51 | 40.16 | 30 | 23.62 | 1 | 6.25 | 9 | 56.25 | 9 | | 56.25 | 9 | | 6.29% | 60 | | 41.96 | 39 | | 27.27 | |
| PROMIS-SRI | 0 | 0 | 51 | 40.16 | 31 | 24.41 | 0 | 0 | 9 | 56.25 | 9 | | 56.25 | 0 | | 0.00% | 60 | | 41.96 | 40 | | 27.97 | |
| SDS | 19 | 14.96 | 55 | 43.31 | 37 | 29.13 | 3 | 18.75 | 10 | 62.5 | 9 | | 56.25 | 22 | | 15.38% | 65 | | 45.45 | 46 | | 32.17 | |
| DSM-5 | 0 | 0 | 51 | 40.16 | 30 | 23.62 | 0 | 0 | 9 | 56.25 | 9 | | 56.25 | 0 | | 0.00% | 60 | | 41.96 | 39 | | 27.27 | |
| *Note.* AIM = Acceptability of Intervention Measure. FIM = Feasibility of Intervention Measure. IAM = Intervention Appropriateness measure. PROMIS-SD = PROMIS Sleep Disruption. PROMIS-SD = PROMIS Sleep Disturbance. PROMIS-SRI = PROMIS Sleep-Related Impairment. DSM-5 = DSM-5 Cross-Cutting. SDS = Sheehan Disability Scale. ‘Standard’ and ‘Adapted’ indicate TSC condition. Pre = pre-treatment assessment. Post = post-treatment assessment. 6FU = six-month follow-up assessment. Each provider only completed the pre-treatment AIM, IAM, FIM one time. They were asked to complete these measures at post for each patient (which is why the post-treatment sample sizes are larger than pre-treatment). Providers were not asked to complete these measures at 6FU. | | | | | | | | | | | | | | | | | | | | | | |  |

**Supplement Table 8. Frequency of PhenX-Assessed Suicidal Behaviors and Illicit Substance Use by Timepoint and TSC Treatment Condition (Standard vs. Adapted)**

|  | **Pre-Treatment** | | **Post-Treatment** | | **6FU** | |
| --- | --- | --- | --- | --- | --- | --- |
|  | **Standard** | **Adapted** | **Standard** | **Adapted** | **Standard** | **Adapted** |
| **Outcome** |  |  |  |  |  |  |
| Actual Suicide Attempts | 0 | 0 | 0 | 0 | 0 | 0 |
| Interrupted Suicide Attempts | 0 | 0 | 0 | 0 | 0 | 0 |
| Aborted Suicide Attempts | 0 | 1 | 0 | 0 | 0 | 0 |
| Preparatory Behaviors for Suicide | 0 | 0 | 0 | 0 | 0 | 0 |
| Non-suicidal self-injury | 0 | 0 | 0 | 0 | 0 | 1 |
| Endorsed using illicit substances | 2 | 39 | 1 | 18 | 2 | 27 |
| *Note.* 6FU = six-month follow-up assessment. ‘Standard’ and ‘Adapted’ indicate TSC condition. | | | | | | |

**Supplement Table 9. Pre-Treatment Patient Demographics by Delayed TSC (UC-DT) compared to Immediate TSC**

| **Characteristic** | **UC-DT (*n* = 65)** | | | |  | | **Immediate (*n* = 78)** | | | |  | |  | |  |
| --- | --- | --- | --- | --- | --- | --- | --- | --- | --- | --- | --- | --- | --- | --- | --- |
|  | ***n*** | | ***%*** | |  | | ***n*** | | ***%*** | | **χ^2^** | | ***p*-value** | |  |
| Sex |  | |  | |  | |  | |  | | 0.31 | | 0.60 | |  |
| Female | 43 | | 66.15 | |  | | 47 | | 60.26 | |  | |  | |  |
| Male | 22 | | 33.85 | |  | | 31 | | 39.74 | |  | |  | |  |
| Ethnicity |  | |  | |  | |  | |  | | 5.30 | | 0.07 | |  |
| Hispanic or Latino | 9 | | 13.85 | |  | | 22 | | 28.21 | |  | |  | |  |
| Not Hispanic or Latino | 55 | | 84.62 | |  | | 56 | | 71.79 | |  | |  | |  |
| Missing/declined to answer | 1 | | 1.54 | |  | | 0 | | 0.00 | |  | |  | |  |
| Race |  | |  | |  | |  | |  | | 6.30 | | 0.40 | |  |
| American Indian/Alaska Native | 1 | | 1.54 | |  | | 5 | | 6.41 | |  | |  | |  |
| Native Hawaiian/Pacific Islander | 2 | | 3.08 | |  | | 0 | | 0.00 | |  | |  | |  |
| Asian | 9 | | 13.85 | |  | | 6 | | 7.69 | |  | |  | |  |
| Black or African American | 14 | | 21.54 | |  | | 15 | | 19.23 | |  | |  | |  |
| White | 24 | | 36.92 | |  | | 34 | | 43.59 | |  | |  | |  |
| More than one race | 9 | | 13.85 | |  | | 12 | | 15.39 | |  | |  | |  |
| Category not listed | 4 | | 6.15 | |  | | 6 | | 7.69 | |  | |  | |  |
| Education |  | |  | |  | |  | |  | | 2.80 | | 0.60 | |  |
| High school graduate or below | 5 | | 7.69 | |  | | 9 | | 11.54 | |  | |  | |  |
| Some or completed college or vocational school | 38 | | 58.46 | |  | | 49 | | 62.82 | |  | |  | |  |
| Some or completed graduate school | 20 | | 23.08 | |  | | 18 | | 23.08 | |  | |  | |  |
| Other | 1 | | 1.54 | |  | | 0 | | 0.00 | |  | |  | |  |
| Missing/declined to answer | 1 | | 1.54 | |  | | 2 | | 2.56 | |  | |  | |  |
| Employment |  | |  | |  | |  | |  | | 2.60 | | 0.50 | |  |
| Full-time | 14 | | 21.54 | |  | | 14 | | 17.95 | |  | |  | |  |
| Part-time | 10 | | 15.34 | |  | | 12 | | 15.34 | |  | |  | |  |
| Not employed | 36 | | 55.34 | |  | | 50 | | 64.10 | |  | |  | |  |
| Other | 5 | | 7.69 | |  | | 2 | | 2.56 | |  | |  | |  |
| Civil Status |  | |  | |  | |  | |  | | 2.80 | | 0.40 | |  |
| Partnered | 11 | | 16.92 | |  | | 17 | | 21.79 | |  | |  | |  |
| Unpartnered | 52 | | 80.00 | |  | | 61 | | 78.21 | |  | |  | |  |
| Other | 1 | | 1.54 | |  | | 0 | | 0.00 | |  | |  | |  |
| Missing/declined to answer | 1 | | 1.54 | |  | | 0 | | 0.00 | |  | |  | |  |
| Living Arrangement | |  | |  | |  | |  | |  | | 2.40 | | 0.70 | |
| Alone | | 11 | | 16.92 | |  | | 16 | | 20.51 | |  | |  | |
| With family | | 38 | | 58.46 | |  | | 43 | | 55.13 | |  | |  | |
| With friend or roommate or pet | | 8 | | 12.31 | |  | | 14 | | 17.95 | |  | |  | |
| Supported housing | | 6 | | 9.23 | |  | | 4 | | 5.13 | |  | |  | |
| Other/category not listed | | 2 | | 3.08 | |  | | 1 | | 1.28 | |  | |  | |
| Government Assistance^a^ | |  | |  | |  | |  | |  | | 3.20 | | 0.90 | |
| Unemployment | | 1 | | 1.54 | |  | | 2 | | 2.56 | |  | |  | |
| Medicare | | 3 | | 4.62 | |  | | 9 | | 11.54 | |  | |  | |
| Medicaid | | 20 | | 30.77 | |  | | 29 | | 37.18 | |  | |  | |
| Social Security | | 7 | | 10.77 | |  | | 9 | | 11.54 | |  | |  | |
| Food Stamps | | 14 | | 21.54 | |  | | 16 | | 20.51 | |  | |  | |
| SSI/SSDI | | 10 | | 15.38 | |  | | 17 | | 21.79 | |  | |  | |
| SNAP | | 5 | | 7.69 | |  | | 9 | | 11.54 | |  | |  | |
| Missing/declined to answer | | 21 | | 32.31 | |  | | 22 | | 28.21 | |  | |  | |
| Annual Personal Income | |  | |  | |  | |  | |  | | 3.80 | | 0.80 | |
| <$10,000 | | 20 | | 30.77 | |  | | 19 | | 24.36 | |  | |  | |
| $10,000-$20,000 | | 15 | | 23.08 | |  | | 19 | | 24.36 | |  | |  | |
| $20,000-$30,000 | | 5 | | 7.69 | |  | | 5 | | 6.41 | |  | |  | |
| $30,00-$40,000 | | 3 | | 4.62 | |  | | 7 | | 8.97 | |  | |  | |
| $40,000-$50,000 | | 3 | | 4.62 | |  | | 5 | | 6.41 | |  | |  | |
| >= $50,000 | | 10 | | 15.38 | |  | | 8 | | 10.26 | |  | |  | |
| I don’t know my income | | 9 | | 13.85 | |  | | 14 | | 17.95 | |  | |  | |
| Missing/declined to answer | | 0 | | 0.00 | |  | | 1 | | 1.28 | |  | |  | |
| Annual Household income | |  | |  | |  | |  | |  | | 8.10 | | 0.30 | |
| <$10,000 | | 13 | | 20.00 | |  | | 8 | | 10.26 | |  | |  | |
| $10,000-$20,000 | | 10 | | 15.38 | |  | | 17 | | 21.79 | |  | |  | |
| $20,000-$30,000 | | 6 | | 9.23 | |  | | 9 | | 11.54 | |  | |  | |
| $30,00-$40,000 | | 2 | | 3.08 | |  | | 5 | | 6.41 | |  | |  | |
| $40,000-$50,000 | | 2 | | 3.08 | |  | | 5 | | 6.41 | |  | |  | |
| >= $50,000 | | 18 | | 27.69 | |  | | 14 | | 17.95 | |  | |  | |
| I don’t know my income | | 14 | | 21.54 | |  | | 18 | | 23.08 | |  | |  | |
| Missing/declined to answer | | 0 | | 0.00 | |  | | 2 | | 2.56 | |  | |  | |
| Self-reported diagnosis^b^ |  | |  | |  | |  | |  | | 7.68 | | 0.66 | |  |
| Neurodevelopmental disorders | 11 | | 16.92 | |  | | 11 | | 14.10 | |  | |  | |  |
| Psychosis | 13 | | 20.00 | |  | | 23 | | 29.49 | |  | |  | |  |
| Mood Disorder Features (Bipolar) | 9 | | 13.85 | |  | | 16 | | 20.51 | |  | |  | |  |
| Mood Disorder Features (Unipolar) | 31 | | 47.69 | |  | | 35 | | 44.87 | |  | |  | |  |
| Anxiety disorders | 39 | | 60.00 | |  | | 33 | | 42.31 | |  | |  | |  |
| Obsessive-compulsive and related disorders | 2 | | 3.08 | |  | | 3 | | 3.85 | |  | |  | |  |
| Trauma and stressor-related disorders | 18 | | 27.69 | |  | | 15 | | 19.23 | |  | |  | |  |
| Dissociative disorders | 0 | | 0.00 | |  | | 0 | | 0.00 | |  | |  | |  |
| Personality disorders | 0 | | 0.00 | |  | | 2 | | 2.56 | |  | |  | |  |
| Feeding and eating disorders | 0 | | 0.00 | |  | | 0 | | 0.00 | |  | |  | |  |
| Substance-related and addictive disorders | 2 | | 3.08 | |  | | 3 | | 3.85 | |  | |  | |  |
| Other/category not listed | 2 | | 3.08 | |  | | 1 | | 1.28 | |  | |  | |  |
| Missing/declined to answer | 7 | | 10.77 | |  | | 8 | | 10.26 | |  | |  | |  |
|  | ***Mean*** | | ***SD*** | |  | | ***Mean*** | | ***SD*** | | **t** | | ***p*-value** | |  |
| Age | 43.78 | | 11.99 | |  | | 43.31 | | 15.07 | | 0.21 | | 0.80 | |  |
| Education (years) | 14.60 | | 3.86 | |  | | 15.04 | | 3.43 | | -0.70 | | 0.50 | |  |
| ^a^Some patients endorsed more than one government assistance category ^b^Comorbidity was common. *Note.* Chi-squared was used for categorical variables, and *t* tests were used for continuous variables. | | | | | | | | | | | | | | |  |

**Supplement Table 10. Means, Standard Deviations, and Effect Sizes for Patient Exploratory Outcomes**

|  | Pre-Treatment | | | | Post-Treatment | | | | 6FU | | | | |  |  |
| --- | --- | --- | --- | --- | --- | --- | --- | --- | --- | --- | --- | --- | --- | --- | --- |
|  | **Standard** | | **Adapted** | | **Standard** | | **Adapted** | | **Standard** | | | **Adapted** | | ***dpre-post*** | ***dpre-6FU*** |
|  | **Mean** | **SD** | **Mean** | **SD** | **Mean** | **SD** | **Mean** | **SD** | | **Mean** | **SD** | **Mean** | **SD** |  |  |
| **Outcome** |  |  |  |  |  |  |  |  | |  |  |  |  |  |  |
| Suicidal Ideation Severity | 0.38 | 0.81 | 0.36 | 0.72 | 0.57 | 1.13 | 0.51 | 0.9 | | 0.43 | 1.13 | 0.38 | 0.83 | -0.03 | -0.03 |
| Mean Caffeinated Drinks Per Day | 2.13 | 1.06 | 1.66 | 1.81 | 2.14 | 2.27 | 1.49 | 1.57 | | 1.33 | 1.37 | 1.92 | 2.53 | -0.10 | 0.90 |
| Mean Days of Alcohol Use in Past 30 Days | 0.19 | 0.54 | 3.34 | 6.05 | 0.14 | 0.38 | 2.55 | 5.17 | | 1.57 | 2.82 | 2.14 | 4.96 | -0.04 | -2.75 |
| Credibility | - | - | - | - | 8.00 | 0.54 | 7.33 | 1.50 | | - | - | - | - | - | - |
| Perceived Improvement | - | - | - | - | 67.14 | 22.89 | 55.07 | 31.19 | | - | - | - | - | - | - |
| *Note.* 6FU = six-month follow-up assessment. "-" means that the variable is not relevant at a given timepoint. ‘Standard’ and ‘Adapted’ indicate TSC condition. Effect sizes are represented with ‘*d*’ and were calculated following Feingold (2009, equation 5), using unadjusted change scores and raw standard deviations at pre-treatment from each treatment condition. Mean cigarettes per day was only reported by patients who endorsed smoking on ‘some days’ or ‘every day.’ | | | | | | | | | | | | | | | |

**Supplement Table 11. Exploratory Aim 1: Multilevel Modeling Results for TSC Condition (Standard vs. Adapted) on Suicidality and Tobacco, Caffeine, and Alcohol Use**

|  | **Post-Treatment** | | | **6FU** | | |
| --- | --- | --- | --- | --- | --- | --- |
|  | ***b*** | **SE** | ***p-*value** | ***b*** | **SE** | ***p-*value** |
| **Outcome** |  |  |  |  |  |  |
| SI Severity | 0.09 | 0.33 | 0.78 | -0.04 | 0.33 | 0.90 |
| Mean Caffeinated Drinks Per Day | -0.40 | 0.74 | 0.59 | 0.58 | 0.78 | 0.46 |
| Mean Days of Alcohol Use in Past 30 Days | -0.42 | 1.65 | 0.80 | -2.01 | 1.64 | 0.22 |
| *Note.* 6FU = six-month follow-up assessment. Bold indicates significant *p*-values. *b* = time-by-treatment interaction. SE = standard errors. Mean cigarettes per day was only reported by patients who endorsed smoking on ‘some days’ or ‘every day.’ When using robust SEs (vs. REML & Kenward-Roger) both with and without county as a covariate there was a marginally significant effect of TSC Condition (Adapted vs. Standard) on change in alcohol use from pre-treatment to 6FU (*b* = -2.01, *SE* = 1.09, *p* = 0.06). | | | | | | |

**Supplement Table 12. Exploratory Aim 2: Multilevel Modeling Moderation Results for UC-DT versus TSC on Patient Outcomes from Pre- to Post-treatment**

|  | ***b*** | **SE** | ***p-*value** |
| --- | --- | --- | --- |
| **Moderator: Sex** |  |  |  |
| PROMIS-SD | 1.01 | 3.13 | 0.75 |
| PROMIS-SRI | -1.93 | 4.01 | 0.63 |
| SHC | 0.16 | 0.61 | 0.80 |
| DSM-5 | -3.03 | 2.88 | 0.29 |
| SDS | 2.72 | 2.74 | 0.32 |
| **Moderator: Age** |  |  |  |
| PROMIS-SD | -2.97 | 3.38 | 0.38 |
| PROMIS-SRI | -5.98 | 4.19 | 0.15 |
| SHC | 0.89 | 0.67 | 0.19 |
| DSM-5 | -0.73 | 2.72 | 0.79 |
| SDS | -0.64 | 2.74 | 0.82 |
| **Moderator: Baseline PROMIS-SD** | | |  |
| PROMIS-SD | - | - | - |
| PROMIS-SRI | -0.02 | 0.30 | 0.95 |
| SHC | -0.06 | 0.04 | 0.10 |
| DSM-5 | -0.12 | 0.17 | 0.49 |
| SDS | -0.35 | 0.21 | 0.10 |
| **Moderator: Baseline PROMIS-SRI** | | |  |
| PROMIS-SD | -0.11 | 0.18 | 0.54 |
| PROMIS-SRI | - | - | - |
| SHC | -0.06 | 0.04 | 0.12 |
| DSM-5 | -0.06 | 0.19 | 0.76 |
| SDS | -0.26 | 0.14 | 0.06 |
| **Moderator: Baseline DSM-5 Cross-Cutting** | | |  |
| PROMIS-SD | -0.15 | 0.17 | 0.37 |
| PROMIS-SRI | -0.01 | 0.22 | 0.96 |
| SHC | -0.02 | 0.03 | 0.45 |
| DSM-5 | - | - | - |
| SDS | -0.11 | 0.15 | 0.48 |
| Note. "-" means that a moderator/outcome pairing was not tested (i.e., when the moderator was already included as the outcome). *b* = time-by-treatment-by-moderator 3-way interaction. SE = robust standard errors. PROMIS-SD = PROMIS Sleep Disruption. PROMIS-SD = PROMIS Sleep Disturbance. PROMIS-SRI = PROMIS Sleep-Related Impairment. SHC = Sleep Health Composite (scored such that higher scores indicate better sleep health). DSM-5 = DSM-5 Cross-Cutting. SDS = Sheehan Disability Scale. | | | |

**Additional File 2: Further information on treatments delivered (also in 10)**

**Transdiagnostic Intervention for Sleep and Circadian Dysfunction (TSC)**

TSC is considered to be “transdiagnostic” in two ways (21): it targets a variety of sleep and circadian diagnoses commonly experienced by individuals diagnosed with a range of SMIs, and it offers a single, brief protocol to address sleep and circadian problems that don't fit neatly into specific categories. TSC was designed to promote sleep health along the six dimensions specified in the Sleep Health Framework (22). Although most providers delivered TSC via individual sessions, some opted to deliver it in a group setting. Note that TSC was originally developed in English, then translated into Spanish to expand access. TSC was offered by 7 Spanish-speaking providers to 7 Spanish-speaking patients. Also, one provider translated the sessions to Vietnamese for 3 patients.

**Standard TSC**

“Standard” TSC is a modular approach, comprised of (a) four core modules that form the basic building blocks of sleep health, (b) four cross-cutting interventions used in every session (e.g., motivational enhancement), and (c) seven optional modules that can be integrated based on case conceptualization, patient goals, and clinical judgment. “Standard” TSC involves relatively high-intensity procedures like other EBPTs, including eight weekly, 50-minute sessions

The *cross-cutting modules* are case formulation, sleep and circadian education, motivational enhancement, and goal setting. *Core module 1* targets irregular sleep-wake times, difficulty winding-down, and difficulty waking-up. *Core module 2* aims to reduce daytime impairment*.* *Core module 3* focuses on unhelpful beliefs about sleep*. Core module 4* aims to promote maintenance of changes made during treatment. *Optional module 1* addresses poor sleep efficiency via stimulus control (23) and sleep restriction (24). *Optional module 2* helps patients reduce time in bed. *Optional module 3* addresses delayed or advanced phase problems (e.g., going to sleep later than desired or waking up earlier than desired). *Optional module 4* helps patients manage worries about sleep. *Optional Module 5* promotes compliance with Continuous Positive Airways Pressure (CPAP) for patients with sleep apnea. *Optional Module 6* helps patients negotiate sleep in complicated environments (e.g., noise from bed/roommates, traffic noise, streetlight entering the bedroom). *Optional Module 7* is for patients who experience nightmares.

**Adapted TSC**

As described in the protocol paper (1), there have been calls for rigorous approaches to treatment adaptation (25-27). In response, we grounded the process for adapting TSC in theory, data, and end-user input. As the overarching guide for the adaptation process, the Replicating Effective Programs (REP) framework (28) was used. Phase 1 of REP (Pre-Condition) was completed prior to the present protocol. First, as discussed in the Introduction of this paper, we established that there is a need for effective, feasible EBPTs for SMI in CMHCs and that sleep and circadian functioning may represent a powerful target to help address this need. Second, we determined that there was empirical support for TSC in CMHCs (29). Third, we gathered end-user input on fit and packaging of the intervention (30, 31). Fourth, we reviewed past data and identified the TSC treatment skills that were most utilized by patients with a utilization scale adapted from Gumport et al. (32). Fifth, we considered TSC’s theoretical underpinnings and mechanisms of action (21, 22) from which we retained the core elements (25, 26, 33). Sixth, we piloted Adapted TSC with 21 adults through the PI’s UC Berkeley research clinic (unpublished data). Informal feedback was solicited from providers and patients who participated in this pilot to further refine Adapted TSC. In Phase 2 of REP (Pre-Implementation), we customized the delivery of TSC training and treatment materials to the CMHC context based on the input from CMHC leadership, staff, and patients (Armstrong et al., 2022; Gumport et al., 2020). Throughout REP Phases 1 and 2, following leading adaptation frameworks, we sought to ensure that Adapted TSC would be relevant to the broadest range of patients and to account for factors that impact implementation (e.g., the resources required) (26, 34, 35). The present trial addressed the last two phases of REP – namely, Phases 3 (Implementation) and 4 (Maintenance and Evolution).

TSC was delivered by CMHC staff across four, 20-minute, weekly sessions. Treatment comprised of the same four *cross-cutting modules* as described for Standard TSC. These were used in all sessions. In addition, the same core modules as Standard TSC were delivered, except that Unhelpful Beliefs about Sleep is no longer included. This is a clarification of our protocol papers for this study (Callaway et al., 2023; Sarfan et al., 2023) in which we stated that the core modules were the same as Standard, but split up into five, rather than four modules. Cosmetically, as the project evolved, we relabeled the core module numbers in the Adapted condition as “1, 2 and 3” versus the Standard labels of “1a, 1b, and 1c” to help the providers follow that these were separate modules to be delivered in different sessions. The one *optional module* focused on reducing sleep-related worry and can be integrated with the core modules, based on clinical presentation, treatment goals, and provider case conceptualization.

***Usual Care and Delayed Treatment with TSC (UC-DT)***

The decision to include UC-DT as the comparison condition was made based on advice from the early CMHC partners to strike a balance between (a) including a comparison group to demonstrate the effectiveness of TSC in community settings; (b) ensuring that *all* participants receive what we hypothesize to be an active treatment (TSC); and (c) maximizing efficiency in terms of study duration, budget, and participants’ time investment. Notably, usual care has been the comparison group in several influential studies (36, 37).

If necessary, within usual care, the provider may refer the patient to additional services, including healthcare, housing support, nutrition, vocational specialists, or peer advocacy. In some cases, patients receive care from interdisciplinary or residential teams, ensuring coordination across multiple service providers.

**Additional File 3: Further information on procedure and measures (also in 10)**

Providers, patients and trainers were consented by the assessment team prior to participation. Participants were told that they could withdraw from the study at any time. Patients were compensated for their participation, and providers were compensated if permitted by their CMHC. Providers were able to bill their time when delivering TSC. Local trainers volunteered to become trainers and were not compensated, however a certification in TSC training and a mug were provided if the trainer trained a minimum of 15 people across at least two trainings and supervised a minimum of three TSC cases*.*

To train Generation 1 providers to be local trainers, the expert trainer first led a 30-minute welcome meeting to provide an overview of the process and offer training in public speaking. Next, the TSC training material was condensed into ‘big picture’ concepts and the content was divided into one-hour chunks and delivered to groups of providers. The expert trainer then conducted “booster trainings” for local trainers to review each content chunk (4-5 boosters for Adapted TSC, 6-7 boosters for Standard TSC). Between booster trainings, the expert trainer offered 30-60 minute 1-on-1 consultations for each trainee*.*

Note that the vast majority of providers and patients were employed by or seeking/receiving treatment at CMHCs. However, in very few isolated instances, providers and patients outside of CMHCs learned about the study (e.g., by word of mouth) and requested to participate. When the providers or patients otherwise met the criteria, they were permitted to participate, and were matched with a CMHC patient or provider, respectively, by the facilitation team.

**Measures**

Detailed information about the measures is included below. .

***Generation 2 Patients***

***Sleep Disturbance.*** The 8-item PROMIS-Sleep Disturbance (PROMIS-SD) assessed disruption to sleep (e.g., trouble staying asleep) over the past seven days. Items were rated on a scale from 1 (not at all/never/very poor) to 5 (very much/always/very good). T-scores were used (Yu et al., 2011), calculated by summing the raw scores and using conversion tables on [healthmeasures.net](http://healthmeasures.net/), where higher scores indicate more severe symptoms. This measure has demonstrated acceptable reliability and validity (38, 39). This was the primary outcome for the patient-level analyses.

***Sleep-Related Impairment.*** The 8-item^[[2]](#footnote-2)^ PROMIS-Sleep Related Impairment (PROMIS-SRI) assessed daytime impairment related to sleep problems using the same scale, timeframe and scoring approach as the PROMIS-SD.

***Functional Impairment.*** Functional impairment was assessed via the Sheehan Disability Scale (SDS) (40). Impairment in work and school, social life, and home and family were rated via three items on a scale from 0 (not at all) to 10 (extremely). Scores ranged from 0-30, with higher scores indicating greater impairment. This measure has demonstrated good reliability and validity (40).

***Overall Sleep Health****.* The Sleep Health Composite measured overall sleep health for the complexity of sleep and circadian problems experienced by people diagnosed with SMI and that are covered by TSC (41). It is defined as the sum of scores on the six sleep health dimensions of the Sleep Health Framework (22) (each dimension dichotomized as 1 = good; 0 = poor): Regularity (midpoint fluctuation), Timing (mean midpoint), Efficiency (sleep efficiency), Duration (total sleep time), Satisfaction (sleep quality question on PROMIS-SD), and Alertness (daytime sleepiness question on PROMIS-SRI). All dimensions – except Satisfaction and Alertness – were assessed via questions about sleep-wake patterns over the past seven days. Scores ranged from 0-6, with higher scores indicating better sleep health. The initial validity of this measure has been established (41).

***Psychiatric Symptoms****.* The DSM-5 Cross-Cutting Measure assessed psychiatric symptoms across 13 mental health domains (e.g., depression, anger, mania, psychosis, substance use). Participants rated how often they were bothered by each symptom on a scale from 0 (not at all) to 4 (nearly every day). Scores ranged from 0-52, with higher scores indicating more severe symptoms. This measure has demonstrated good test-retest reliability and clinical utility (42, 43).

***PhenX Toolkit.*** Scales from the PhenX Toolkit (44) were used to assess various patient outcomes. To assess suicidal ideation and behaviors, two subscales from the screening version of the Columbia-Suicide Severity Rating Scale—Severity of Suicidal Ideation and Suicidal Behavior—were administered. Ideation was assessed in the past month and suicidal behavior in the past three months. These scales were scored according to the scoring guide (45). For ideation, the highest numerical value (i.e., the value associated with the most severe item endorsed, ranging from 1 to 5) was used as the final score. For suicidal behavior, five suicide-related behaviors were assessed by separate items, scored with a binary scale (0 = no, 1 = yes) and frequency of patients who endorsed a given behavior was identified. The PhenX ‘Alcohol – 30-Day Quantity and Frequency’, ‘Tobacco – 30 Day Quantity and Frequency’, ‘Substances – 30-Day Frequency’, and ‘Supplemental Beverage Questionnaire’ were used to assess alcohol, tobacco, psychoactive substance, and caffeine consumption over the past 30 days. As described below, too few participants reported suicidal behavior or used tobacco. Thus, these variables were not analyzed.

To assess suicidal ideation and behaviors, the above scales were scored according to the scoring guide (45).

To ease patient burden, this measure was adapted slightly, such that if patients denied suicidal ideation, they were not required to answer questions about suicidal behavior. These scales were scored according to the scoring guide (45). Specifically, the suicidal ideation scale was scored such that each question was assigned a sequential, numerical value ranging from 1 to 5 as the questions increased in severity (Question 1: “Have you wished you were dead or wished you could go to sleep and not wake up?” = 1, to Question 5: "Have you started to work out or worked out the details of how to kill yourself? Do you intend to carry out this plan?” = 5). The highest numerical value (i.e., the value associated with the most severe item endorsed) was used as the final score. Participants were given a 0 if no ideation was endorsed. For suicidal behavior, participants were asked whether they had engaged in five suicide-related behaviors in the past three months (i.e., actual attempt, aborted attempt, interrupted attempt, preparatory behavior/s, and/or nonsuicidal self-injury). Each item was reported on a binary scale (0 = no, 1 = yes) and frequency of patients who endorsed a given behavior was identified.

To assess alcohol, select questions from the PhenX ‘Alcohol - 30-Day Quantity and Frequency’ protocol were administered. Following prior research (e.g., 46), number of days on which patients drank alcohol in the past 30 days was used as the outcome.

To assess tobacco, the PhenX ‘Tobacco - 30-Day Quantity and Frequency - Adult' protocol was used. This measure has three sets of question protocols: (1) a protocol for ‘Every-Day Smokers,’ (2) a protocol for ‘Some-Day Smokers,’ and (3) a protocol for ‘Former Smokers.’ If patients reported that they had never smoked tobacco, this measure was skipped. Following prior research, the outcome computed for the present study was average cigarettes smoked per day (CPD) (e.g., 47). Specifically, if patients reported that they smoked ‘every day’ at the time of assessment, they were asked to report the average number of cigarettes that they smoked per day. If patients reported that they smoked on ‘some days’ at the time of assessment, they were asked to report the number of days they smoked cigarettes in the past 30 days and, on average on those days, how many cigarettes they smoked. CPD was computed across these two groups of patients.

Caffeine was assessed using questions adapted from the ‘Supplemental Beverage Questionnaire.’ The original measure suggested calculating the caffeine milligrams ingested per year (48). However, to reduce participant burden and better capture the timeframes assessed in the present study, some questions from the original measure were not administered – in particular, the type of caffeinated drink consumed (used to calculate annual caffeine servings in the original measure). Together, number of caffeinated drinks per day, on average, over the past 30 days was used as the outcome.

To assess use of other psychoactive substances, the PhenX ‘Substances - 30-Day Frequency’ protocol was used. This measure assesses the number of days on which participants used substances such as sedatives, painkillers, stimulants, and hallucinogens over the past 30 days. Following past research (49-51), a binary variable was created by collapsing across substances to indicate use of any of these substances over the past 30 days (0 = no use of a psychoactive substance over the past 30 days, 1 = use of a psychoactive substance over the past 30 days).

***Credibility and Perceived Improvement*.** At the post-treatment assessment, perceptions of TSC’s credibility and symptom improvement were assessed by four questions adapted from the Credibility/Expectancy Questionnaire (CEQ) (Devilly & Borkovec, 2000). These questions assessed (1) how logical TSC seemed, (2) how successful it was in reducing sleep symptoms, (3) how confident patients would be in recommending TSC to a friend, and (4) how much improvement patients believe had occurred. All questions were rated on a scale from 0 (not at all) to 9 (very), except for perceived improvement, which was rated as a percentage from 0-100%.

***Generation 2 Providers***

***Acceptability.*** Providers rated the acceptability of TSC via the *Acceptability of Intervention Measure* (AIM) (Weiner et al., 2017). This 4-item measure was rated on a scale from 1 (completely disagree) to 5 (completely agree). This measure has demonstrated satisfactory validity, internal reliability, test-retest reliability, and sensitivity to change (52). This was the primary outcome for the provider-level analyses.

***Appropriateness and Feasibility****.* Providers rated the appropriateness and feasibility of TSC via the following 4-item measures: *Feasibility of Intervention* *Measure* (FIM) and *Intervention Appropriateness Measure* (IAM) (52) using the same scale as the AIM.

***Number of TSC Sessions.*** The number of sessions delivered to each enrolled patient by each provider was counted.

***Occupation***. Providers were asked to report their current position, professional degree, and work history, including their caseload, theoretical orientation, licensure status, and previous training in sleep treatment.

**UC-DT Contamination**

At the end of UC-DT and before starting TSC, we assessed for patient exposure to TSC during the UC-DT waiting period by asking “Have you received any sleep intervention, treatment, or coaching since entering the study?” If yes, the assessor asked for details. Two independent coders rated the responses for potential exposure to TSC. Only six patients reported having potentially received a part of TSC during the UC-DT waiting period (e.g., “covering energy generating and daytime tiredness”, “sleep diary with sleep therapist”). We deemed this to indicate minimal contamination.

**References Cited**

1. Sarfan LD, Agnew ER, Diaz M, Dong L, Fisher K, Spencer JM, et al. The Transdiagnostic Intervention for Sleep and Circadian Dysfunction (TranS-C) for serious mental illness in community mental health part 1: study protocol for a hybrid type 2 effectiveness-implementation cluster-randomized trial. Trials. 2023;24(1):1-18.

2. Callaway CA, Sarfan LD, Agnew ER, Dong L, Spencer JM, Hache RE, et al. The Transdiagnostic Intervention for Sleep and Circadian Dysfunction (TranS-C) for serious mental illness in community mental health part 2: study protocol for a hybrid type 2 effectiveness-implementation cluster-randomized trial using train-the-trainer. Trials. 2023;24(1):503.

3. Raudenbush SW, Spybrook J, Congdon R, Liu X-f, Martinez A, Bloom H, et al. Optimal design software for multi-level and longitudinal research (Version 3.01)[Software]. Retrieved from. 2011.

4. Spybrook J, Bloom H, Congdon R, Hill C, Martinez A, Raudenbush S. Optimal design for longitudinal and multilevel research: Documentation for the optimal design software (Version 3.0). Retrieved from <www.wtgrantfoundation.org> 2011 [

5. Black AC, Harel O, Betsy McCoach D. Missing data techniques for multilevel data: Implications of model misspecification. Journal of Applied Statistics. 2011;38(9):1845-65.

6. Bell BA, Morgan GB, Schoeneberger JA, Kromrey JD, Ferron JM. How low can you go? An investigation of the influence of sample size and model complexity on point and interval estimates in two-level linear models. Methodology: European Journal of Research Methods for the Behavioral and Social Sciences. 2014;10 (1):1-11.

7. McNeish DM, Stapleton LM. The effect of small sample size on two-level model estimates: A review and illustration. Educational Psychology Review. 2016;28:295-314.

8. Feingold A. Effect sizes for growth-modeling analysis for controlled clinical trials in the same metric as for classical analysis. Psychological Methods. 2009;14:43.

9. Benjamini Y, Hochberg Y. Controlling the false discovery rate: a practical and powerful approach to multiple testing. Journal of the Royal statistical society: series B (Methodological). 1995;57(1):289-300.

10. Harvey AG, Agnew ER, Esteva Hache R, Spencer JM, Diaz M, Patino EO, et al. A randomized trial of Standard versus Adapted versions of the Transdiagnostic Intervention for Sleep and Circadian dysfunction implemented via facilitation and delivered by community mental health providers: Improving the “fit” of psychological treatments by adapting to context. Mauscript submitted for publication. in press.

11. Richardson JT. Eta squared and partial eta squared as measures of effect size in educational research. Educational research review. 2011;6(2):135-47.

12. Valente MJ, MacKinnon DP. Comparing models of change to estimate the mediated effect in the pretest–posttest control group design. Structural equation modeling: a multidisciplinary journal. 2017;24(3):428-50.

13. Fishbein JN, Judd CM, Genung S, Stanton AL, Arch JJ. Intervention and mediation effects of target processes in a randomized controlled trial of Acceptance and Commitment Therapy for anxious cancer survivors in community oncology clinics. Behaviour Research and Therapy. 2022;153:104103.

14. Goldsmith KA, MacKinnon DP, Chalder T, White PD, Sharpe M, Pickles A. Tutorial: The practical application of longitudinal structural equation mediation models in clinical trials. Psychological methods. 2018;23(2):191.

15. Lee H, Cashin AG, Lamb SE, Hopewell S, Vansteelandt S, VanderWeele TJ, et al. A guideline for reporting mediation analyses of randomized trials and observational studies: the AGReMA statement. Jama. 2021;326(11):1045-56.

16. Gelman A, Hill J. Data analysis using regression and multilevel/hierarchical models: Cambridge university press; 2006.

17. Dolsen MR, Harvey AG. Life‐time history of insomnia and hypersomnia symptoms as correlates of alcohol, cocaine and heroin use and relapse among adults seeking substance use treatment in the United States from 1991 to 1994. Addiction. 2017;112(6):1104-11.

18. Klumpp H, Chang F, Bauer BW, Burgess HJ. Objective and subjective sleep measures are related to suicidal ideation and are transdiagnostic features of major depressive disorder and social anxiety disorder. Brain sciences. 2023;13(2):288.

19. Armstrong CC, Dong L, Harvey AG. Mediators and moderators of outcome from the Transdiagnostic Sleep and Circadian Intervention for adults with severe mental illness in a community setting. Behaviour Research and Therapy. 2022:104053.

20. Waters F, Chiu VW, Dragovic M, Ree M. Different patterns of treatment response to Cognitive-Behavioural Therapy for Insomnia (CBT-I) in psychosis. Schizophrenia Research. 2020;221:57-62.

21. Harvey AG, Buysse DJ. Treating Sleep Problems: A Transdiagnostic Approach: Guilford Publications; 2017.

22. Buysse DJ. Sleep health: can we define it? Does it matter? Sleep. 2014;37(1):9-17.

23. Bootzin RR. Stimulus control treatment for insomnia. Proceedings of the American Psychological Association. 1972;7:395-6.

24. Spielman AJ, Caruso LS, Glovinsky PB. A behavioral perspective on insomnia treatment. Psychiatric Clinics of North America. 1987;10:541-53.

25. Escoffery C, Lebow-Skelley E, Haardoerfer R, Boing E, Udelson H, Wood R, et al. Systematic review of adaptations of public health evidence-based interventions. Implementation Science. 2018;13(1):125.

26. Escoffery C, Lebow-Skelley E, Udelson H, Böing EA, Wood R, Fernandez ME, et al. A scoping study of frameworks for adapting public health evidence-based interventions. Translational Behavioral Medicine. 2018;9(1):1-10.

27. Harvey AG, Lammers H, Dolsen MR, Tran M, Tuck A, Hilmoe H, et al. Advancing the science of treatment adaptation and ad hoc adaptations of cognitive, behavioral and related evidence-based psychosocial treatments for adults diagnosed with a mental illness: a systematic review. . Evidence-Based Meantl Health. 2020.

28. Kilbourne AM, Neumann MS, Pincus HA, Bauer MS, Stall R. Implementing evidence-based interventions in health care: application of the replicating effective programs framework. Implementation Science. 2007;2(1):1-10.

29. Harvey AG, Dong L, Hein K, Yu S, Martinez A, Gumport N, et al. A randomized controlled trial of the Transdiagnostic Intervention for Sleep and Circadian Dysfunction (TranS-C) to improve serious mental illness outcomes in a community setting. Journal of Consulting and Clinical Psychology. 2021;89(6):537-44.

30. Armstrong CC, Harvey AG. Barriers and facilitators to behavior change for individuals with severe mental illness who received the transdiagnostic intervention for sleep and circadian dysfunction in a community mental health setting. The Journal of Behavioral Health Services & Research. 2021:1017.

31. Gumport NB, Yu SH, Harvey AG. Implementing a transdiagnostic sleep and circadian intervention in a community mental health setting: A qualitative process evaluation with community stakeholders. Psychiatry Research. 2020;293:113443.

32. Gumport NB, Dolsen MR, Harvey AG. Usefulness and Utilization of Treatment Elements from the Transdiagnostic Sleep and Circadian Intervention with Adolescents with an Evening Circadian Preference. Behavior Research and Therapy. 2019;123:103504.

33. Lee SJ, Altschul I, Mowbray CT. Using planned adaptation to implement evidence‐based programs with new populations. American Journal of Community Psychology. 2008;41(3-4):290-303.

34. Aarons GA, Green AE, Palinkas LA, Self-Brown S, Whitaker DJ, Lutzker JR, et al. Dynamic adaptation process to implement an evidence-based child maltreatment intervention. Implementation Science. 2012;7(1):32.

35. Weisz JR. Building robust psychotherapies for children and adolescents. Perspectives on Psychological Science. 2014;9(1):81-4.

36. Weisz JR, Chorpita BF, Palinkas LA, Schoenwald SK, Miranda J, Bearman SK, et al. Testing standard and modular designs for psychotherapy treating depression, anxiety, and conduct problems in youth: A randomized effectiveness trial. Archives of general psychiatry. 2012;69(3):274-82.

37. Craske MG, Stein MB, Sullivan G, Sherbourne C, Bystritsky A, Rose RD, et al. Disorder-specific impact of coordinated anxiety learning and management treatment for anxiety disorders in primary care. Archives of General Psychiatry. 2011;68(4):378-88.

38. Yu L, Buysse DJ, Germain A, Moul D. Development of short forms from the PROMIS sleep disturbance and sleep-related impairment item banks. Behavioral Sleep Medicine. 2012;10:6-24.

39. Buysse DJ, Yu L, Moul DE, Germain A, Stover A, Dodds NE, et al. Development and validation of patient-reported outcome measures for sleep disturbance and sleep-related impairments. Sleep. 2010;33:781-92.

40. Sheehan DV, Harnett-Sheehan K, Raj BA. The measurement of disability. International Clinical Psychopharmacology. 1996;11(suppl 3):89-95.

41. Dong L, Martinez AJ, Buysse DJ, Harvey AG. A composite measure of sleep health predicts concurrent mental and physical health outcomes in adolescents prone to eveningness. Sleep health. 2019;5(2):166-74.

42. Narrow WE, Clarke DE, Kuramoto SJ, Kraemer HC, Kupfer DJ, Greiner L, et al. DSM-5 field trials in the United States and Canada, Part III: development and reliability testing of a cross-cutting symptom assessment for DSM-5. American Journal of Psychiatry. 2013;170(1):71-82.

43. Clarke DE, Kuhl EA. DSM-5 cross-cutting symptom measures: a step towards the future of psychiatric care? World Psychiatry. 2014;13(3):314-6.

44. Hamilton CM, Strader LC, Pratt JG, Maiese D, Hendershot T, Kwok RK, et al. The PhenX Toolkit: get the most from your measures. American journal of epidemiology. 2011;174(3):253-60.

45. Nilsson ME, Suryawanshi S, Gassmann-Mayer C, Dubrava S, McSorley P, Jiang K. Columbia-Suicide Severity Rating Scale Scoring and Analysis Guide (Version 2.0). Retrieved from <https://cssrs.columbia.edu/wp-content/uploads/ScoringandDataAnalysisGuide-for-Clinical-Trials-1.pdf2013>.

46. Hinojosa CA, Liew A, An X, Stevens JS, Basu A, Van Rooij SJ, et al. Associations of alcohol and cannabis use with change in posttraumatic stress disorder and depression symptoms over time in recently trauma-exposed individuals. Psychological medicine. 2024;54(2):338-49.

47. Sakuma K-LK, Pierce JP, Fagan P, Nguyen-Grozavu FT, Leas EC, Messer K, et al. Racial/ethnic disparities across indicators of cigarette smoking in the era of increased tobacco control, 1992–2019. Nicotine and Tobacco Research. 2021;23(6):909-19.

48. Song YJ, Kristal AR, Wicklund KG, Cushing-Haugen KL, Rossing MA. Coffee, tea, colas, and risk of epithelial ovarian cancer. Cancer Epidemiology Biomarkers & Prevention. 2008;17(3):712-6.

49. Brown JL, Cochran G, Bryan MA, Charron E, Winhusen TJ. Associations between elevated depressive symptoms and substance use, prescription opioid misuse, overdose history, pain, and general health among community pharmacy patients prescribed opioids. Substance abuse. 2022;43(1):1110-5.

50. Gerke DR, Call J, Auslander WF. The syndemic factors of violence exposure, Substance Use, and Mental Health problems: Relationships to sexual risk behaviors in HIV-Negative Young Men who have sex with men. Journal of the Society for Social Work and Research. 2022;13(2):235-59.

51. Shmulewitz D, Stohl M, Greenstein E, Roncone S, Walsh C, Aharonovich E, et al. Validity of the DSM-5 craving criterion for alcohol, tobacco, cannabis, cocaine, heroin, and non-prescription use of prescription painkillers (opioids). Psychological medicine. 2023;53(5):1955-69.

52. Weiner BJ, Lewis CC, Stanick C, Powell BJ, Dorsey CN, Clary AS, et al. Psychometric assessment of three newly developed implementation outcome measures. Implementation Science. 2017;12(1):108.

1. This approach to mediation deviates from the approach described in the protocol paper (i.e., multilevel SEMs). This change was made, because there is limited published guidance on using multilevel SEM for mediation in longitudinal RCTs. [↑](#footnote-ref-1)
2. Due to administrative error, the 16-item version was originally administered. However, standardized T-scores are only available for the 8-item version. Thus, only the 8 items that comprise the 8-item version were included in the scoring and analyses. [↑](#footnote-ref-2)
